# Targeting of focal adhesion kinase enhances the immunogenic cell death of PEGylated liposome doxorubicin to optimize therapeutic responses of immune checkpoint blockade

**DOI:** 10.1186/s13046-024-02974-4

**Published:** 2024-02-19

**Authors:** Baoyuan Zhang, Ning Li, Jiaming Gao, Yuxi Zhao, Jun Jiang, Shuang Xie, Cuiping Zhang, Qingyu Zhang, Leo Liu, Zaiqi Wang, Dongmei Ji, Lingying Wu, Ruibao Ren

**Affiliations:** 1 State Key Laboratory for Medical Genomics, Collaborative Innovation Center of Hematology, Shanghai Institute of HematologyNational Research Center for Translational MedicineRuijin Hospital Affiliated to Shanghai Jiao Tong University School of Medicine, Shanghai, China; 2 Department of Gynecologic Oncology, National Cancer Center/National Clinical Research Center for Cancer/Cancer Hospital, Chinses Academy of Medical Sciences and Peking Union Medical College, Beijing, China; 3 Department of Medical Oncology, Fudan University Shanghai Cancer Center, Shanghai, China; 4 International Center for Aging and Cancer, Hainan Medical University, Hainan Province, Haikou, China; 5 InxMed (Shanghai) Co., Ltd, Beijing, China; 6 Department of Pathology, Yantai Affiliated Hospital of Binzhou Medical University, Yantai, Shandong China; 7 Laboratory of Obstetrics and Gynecology, Affiliated Hospital of Guangdong Medical University, Zhanjiang, Guangdong China

**Keywords:** FAK, Pegylated liposome doxorubicin (PLD), Synergy, Immunogenic cell death (ICD), Immune checkpoint blockade (ICB)

## Abstract

**Backgrounds:**

Immune checkpoint blockade (ICB) is widely considered to exert long-term treatment benefits by activating antitumor immunity. However, many cancer patients show poor clinical responses to ICB due in part to the lack of an immunogenic niche. Focal adhesion kinase (FAK) is frequently amplified and acts as an immune modulator across cancer types. However, evidence illustrates that targeting FAK is most effective in combination therapy rather than in monotherapy.

**Methods:**

Here, we used drug screening, in vitro and in vivo assays to filter out that doxorubicin and its liposomal form pegylated liposome doxorubicin (PLD) showed synergistic anti-tumor effects in combination with FAK inhibitor IN10018. We hypothesized that anti-tumor immunity and immunogenic cell death (ICD) may be involved in the treatment outcomes through the data analysis of our clinical trial testing the combination of IN10018 and PLD. We then performed cell-based assays and animal studies to detect whether FAK inhibition by IN10018 can boost the ICD of PLD/doxorubicin and further established syngeneic models to test the antitumor effect of triplet combination of PLD, IN10018, and ICB.

**Results:**

We demonstrated that the combination of FAK inhibitor IN10018, and PLD/doxorubicin exerted effective antitumor activity. Notably, the doublet combination regimen exhibited response latency and long-lasting treatment effects clinically, outcomes frequently observed in immunotherapy. Our preclinical study confirmed that the 2-drug combination can maximize the ICD of cancer cells. This approach primed the tumor microenvironment, supplementing it with sufficient tumor-infiltrating lymphocytes (TILs) to activate antitumor immunity. Finally, different animal studies confirmed that the antitumor effects of ICB can be significantly enhanced by this doublet regimen.

**Conclusions:**

We confirmed that targeting FAK by IN10018 can enhance the ICD of PLD/doxorubicin, further benefiting the anti-tumor effect of ICB. The animal tests of the triplet regimen warrant further discovery in the real world.

**Supplementary Information:**

The online version contains supplementary material available at 10.1186/s13046-024-02974-4.

## Introduction

Chemotherapy and targeted therapy have been proven in effective reduction of tumor burden across cancer types. These treatments utilize direct cancer cell-killing manners which may shortly induce drug resistance to impact the long-term benefits of the cancer patients [1]. Immunotherapies, including immune checkpoints (PD-1/PD-L1, CTLA4, TIGIT, LAG3, and others) blockade (ICB) may overcome the pitfalls of these conventional anticancer drugs and produce prolonged responses through activation of cell-killing lymphocytes within the tumor microenvironment [2]. The first ICB agent, ipilimumab that targets CTLA4 of T cells was approved for melanoma therapy in 2011 [3]. A series of ICB agents have since been developed and launched for cancer treatment. Mechanistically, immune checkpoints can protect cancer cells from cytotoxic T lymphocytes (CTLs). Blockade of these targets can activate the cancer cell-killing effects of CTLs [4]. Unfortunately, some cancers such as ovarian cancer and a few types of gastrointestinal cancer do not respond well to ICB due in part to the lack of tumor-infiltrating lymphocytes (TILs). These cancer types are often termed “cold” tumors [5].

Recent reports indicated that immunogenic cell death (ICD) induced by specific anticancer drugs may further enhance the efficacy of ICB. ICD is a unique cell death pattern in which dying cancer cells release damage-associated molecular patterns (DAMPs) into the tumor microenvironment. DAMPs comprise a series of molecules including calreticulin, high mobility group box protein 1 (HMGB1), annexin A1, GRP94, ATP, and others [6, 7]. These biomarkers facilitate the maturation process of antigen-presenting cells (APCs) in the presence of tumor antigens and sequentially boost the differentiation of naïve T cells into CTLs [8]. PEGylated liposome doxorubicin (PLD) and its active ingredient, doxorubicin are capable of inducing ICD and synergizing with PD-1/PD-L1 blockade in animal studies [9, 10]. PLD tends to sequester the free doxorubicin away from organs such as the heart to avoid cardiovascular toxicity and enrich the drug in tumors to increase the concentration of the active ingredient, enhancing the cancer cell-killing effects. The liposome formulation can also increase the retaining time of the free drug in life, so that low frequency of dosing is enough to maintain the anti-cancer effects [11]. Because of these features, PLD is frequently used instead of free doxorubicin in clinical settings. The doublet regimen of PLD and PD-L1 blockade clinically exhibited an overall response rate (ORR) of 13.3% and median progression-free survival (PFS) of 3.7 months in the treatment of platinum-resistant ovarian cancer. Although this is a significant improvement compared to the drug effects of PD-1/PD-L1 blockade alone, many patients still lack effective responses to this regimen [12].

Focal adhesion kinase (FAK) is a non-tyrosine kinase that chiefly regulates the tumor microenvironment [13], antitumor immunity [14], and cancer stem cells [15]. Elevated expression of FAK is associated with the progression of different cancer types. Multiple FAK inhibitors have been developed, aiming for the treatment of various indications [16]. In addition, activated FAK signaling is recognized as a mechanism for drug resistance of cancer cells. Therefore, FAK inhibition is hypothesized to be effective in combination therapies [17, 18]. Given these characteristics, targeting of FAK is considered to amplify the effects of other cancer drugs in clinical settings [19]. Indeed, a panel of FAK inhibitors are being evaluated in this trend [20].

In the present study, we focused on a clinical-stage small molecule FAK inhibitor IN10018 (previously known as BI853520 [21, 22]), which has obtained fast-track designation from the U.S. Food and Drug Administration (FDA) and breakthrough designation from China National Medical Products Administration (NMPA) for its combination with PLD in the treatment of platinum-resistant ovarian cancer (PROC) (clinical trial ID: NCT05551507). The previous data were reported at the 2022 ASCO annual meeting [23]. Here, we recapitulated the rationale and discovery process in identifying this regimen. We also updated the readout from the trial and the clinical outcome exceeded our expectations based on the preclinical data. As of the cutoff date of 31 May 2022, the phase Ib single-arm study demonstrated an ORR of 54.8% and a median PFS of 7.3 months, sharply elevating the antitumor response from the monotherapy employing only PLD [24]. It is noteworthy that 10 patients exhibiting partial response (PR) were reported as having stable disease (SD) at the first radiographical assessment and gradually became PR following treatment. 1 out of these 10 patients showed pseudoprogression based on the first tumor size evaluation, occasionally observed in immunotherapies, and became PR at week 30 post the dosing initiation. These evidence suggested that antitumor immunity may be involved in the underlying biology of clinical efficacy [25]. Most of our preclinical work was performed with immune-compromised models, partially explaining why preclinical settings may underestimate the outcome of the regimen.

To determine if the observed efficacy was associated with the stimulation of antitumor immunity, we established and performed preclinical assays. We found that IN10018 in combination with PLD or its active ingredient, doxorubicin can enhance antitumor effects, over-presenting ICD hallmarks significantly compared to the monotherapies. The 2-drug combination further strengthened anticancer immunity to maximize the efficacy of ICB. We wish the triplet regimen could provide more therapeutic opportunities for ICB non-responders.

## Results

### Targeting of FAK in combination with anthracyclines exhibits the most potent cancer cell killing through chemotherapeutic drug screening

**Fig. 1:**
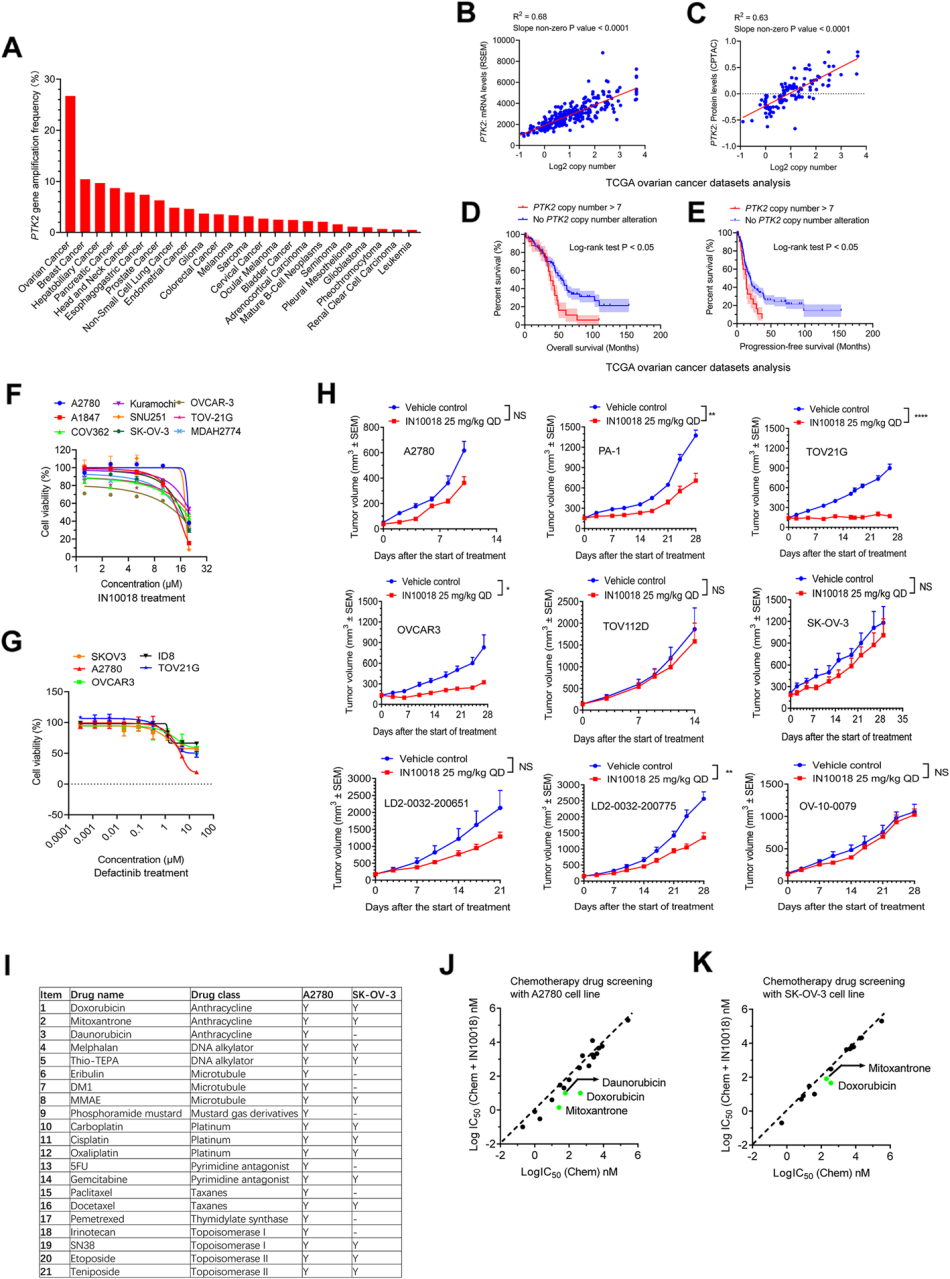
Targeting FAK in combination with anthracyclines exhibits the most potent cancer cell killing through chemotherapeutic drug screening. **A** Patient ratios of copy number amplification across different cancer types from TCGA datasets. **B-C** The correlation analysis between log2 copy numbers and mRNA levels of *PTK2* or FAK protein levels in ovarian cancer from TCGA datasets. R.^2^ and slope non-zero *p* value were obtained to analyze correlation significance. **D-E** The overall and progression-free survival analyses between patients with *PTK2* high amplification (copy number > 7) and without *PTK2* copy number variation for ovarian cancer from TCGA datasets. **F-G** The cell-killing effects of IN10018 or defactinib in the treatment of different ovarian cancer cell lines (*n* = 3 per point). **H** The antitumor effects of IN10018 in the treatment of different ovarian cancer animal models including 6 CDX models and 3 PDX models (*n* = 3 per group). **I** The drug details of the chemotherapeutic drug screening. **J-K** The IC50 comparison between the tested drugs alone (x-axis) or the combination of tested drugs and 3 μM IN10018 (y-axis) in the screening with ovarian cancer cell lines A2780 or SK-OV-3. Data represent mean ± SEM. Log-rank testing was performed for the analysis of statistical significance. The unpaired student's T-test was used for the other statistical analysis. NS means non-significant, *P < 0.05, and **P < 0.01

FAK protein is encoded by the *PTK2* gene, which shows high copy number amplification across different cancer types, indicating that FAK may play an important role in cancer development. From The Cancer Genome Atlas (TCGA) data analysis, we found that *PTK2* exhibits the highest copy number amplification in ovarian cancer compared to other cancer types (Fig. 1A). The copy numbers of the *PTK2* gene are highly correlated with the mRNA expression and protein levels of FAK (Fig. 1B-C). The overall survival data of ovarian cancer in the TCGA project were downloaded and separated into two subgroups based on the copy number values of patients. The results illustrated that the *PTK2* high copy number subgroup (copy number value greater than 7) exhibited a poorer survival profile compared to the *PTK2* non-alteration subgroup (Fig. 1D). Similar results were also found in the analysis of progression-free survival data (Fig. 1E). Moreover, the data from the MD Anderson cell lines project (MCLP) indicated that phospho FAK Y397 (which represents the activity of FAK) is overexpressed in multiple cancer types, especially in ovarian cancer [26]. Therefore, FAK may serve as a meaningful target for cancer treatment (Supplementary Fig. 1A).

Although FAK is frequently amplified and activated across multiple cancer types, the antitumor potency of monotherapy approaches by FAK inhibition remains elusive. To evaluate the treatment effects of FAK inhibition alone, we began with cell-killing tests in ovarian cancer, as disease development positively correlates with FAK function. Unexpectedly, two clinical-stage FAK inhibitors, IN10018, and defactinib had limited cell-killing effects for different ovarian cancer cell lines in vitro (Fig. 1F-G). Moreover, FAK inhibition by PF-573228 did not show improved anticancer effects in ovarian cancer as opposed to the other cancer types from a drug screening provided by the cancer therapeutics response portal (CTRP) (Supplementary Fig. 1B) [27]. This suggested that the limited effects of FAK inhibition are not restrained in only ovarian cancer.

We then explored the potency of tumor growth inhibition by IN10018 with ovarian cancer patient-derived xenograft (PDX) and cell line-derived xenograft (CDX) models. All the used models showed moderate-to-high expression levels of FAK in the tumor tissues, similar to the data from bioinformatics analysis of the human ovarian cancer tissues (Supplementary Fig. 2A-I). As the results of the cell line-based assay, IN10018 only exhibited slight-to-moderate antitumor effects at the animal level (Fig. 1H, Supplementary Fig. 1C-K). Therefore, both in vitro and in vivo tests demonstrated that monotherapy with FAK inhibition may not be sufficient to produce robust efficacy in cancer treatment. However, combination therapy may pave the way for new approaches to overcome the shortcomings from the monotherapy.

To discover effective combination strategies for FAK inhibition, we performed a chemotherapeutic drug screen to test the overall cell-killing effects of conventional chemotherapies with or without supplementation of IN10018 in 2 ovarian cancer cell lines, A2780 and SK-OV-3 (Fig. 1I-K). Most of this drug series tended to receive enhanced impacts from supplemental IN10018. However, the anthracyclines, especially doxorubicin, exhibited the best enhanced antitumor effects in combination with IN10018 amongst all the tested drug series. Interestingly, the drug screening with colorectal cancer cell line CT26 and pancreatic cancer cell line KPC also showed the most tendency on killing the cancer cells by IN10018 plus anthracyclines compared to the other drug series, suggesting that the combination benefits are not restrained to specific cancer types (Supplementary Fig. 1L-N). As doxorubicin and its liposome formulation, PLD are frequently used in clinical therapy for ovarian, breast, lung, and head and neck cancer, this particular combined regimen deserves further exploration in cancer treatment.

### FAK targeting synergizes with doxorubicin/PLD in the treatment of cancer in both preclinical and clinical settings

PLD is frequently used in the clinical setting as opposed to doxorubicin as the compound alone may cause cardiotoxicity due to its overdistribution in the heart. The liposome formulation is able to limit the concentration of doxorubicin in the heart to overcome this adverse effect. PLD is inappropriate for tests in vitro due to its slow release of the active ingredient. Therefore, in this study, PLD was tested in vivo*,* and its active ingredient, doxorubicin was used for in vitro assays. Based on preclinical results, a phase Ib clinical trial was designed as a single-arm study that tested the combined benefit of PLD and IN10018.

**Fig. 2:**
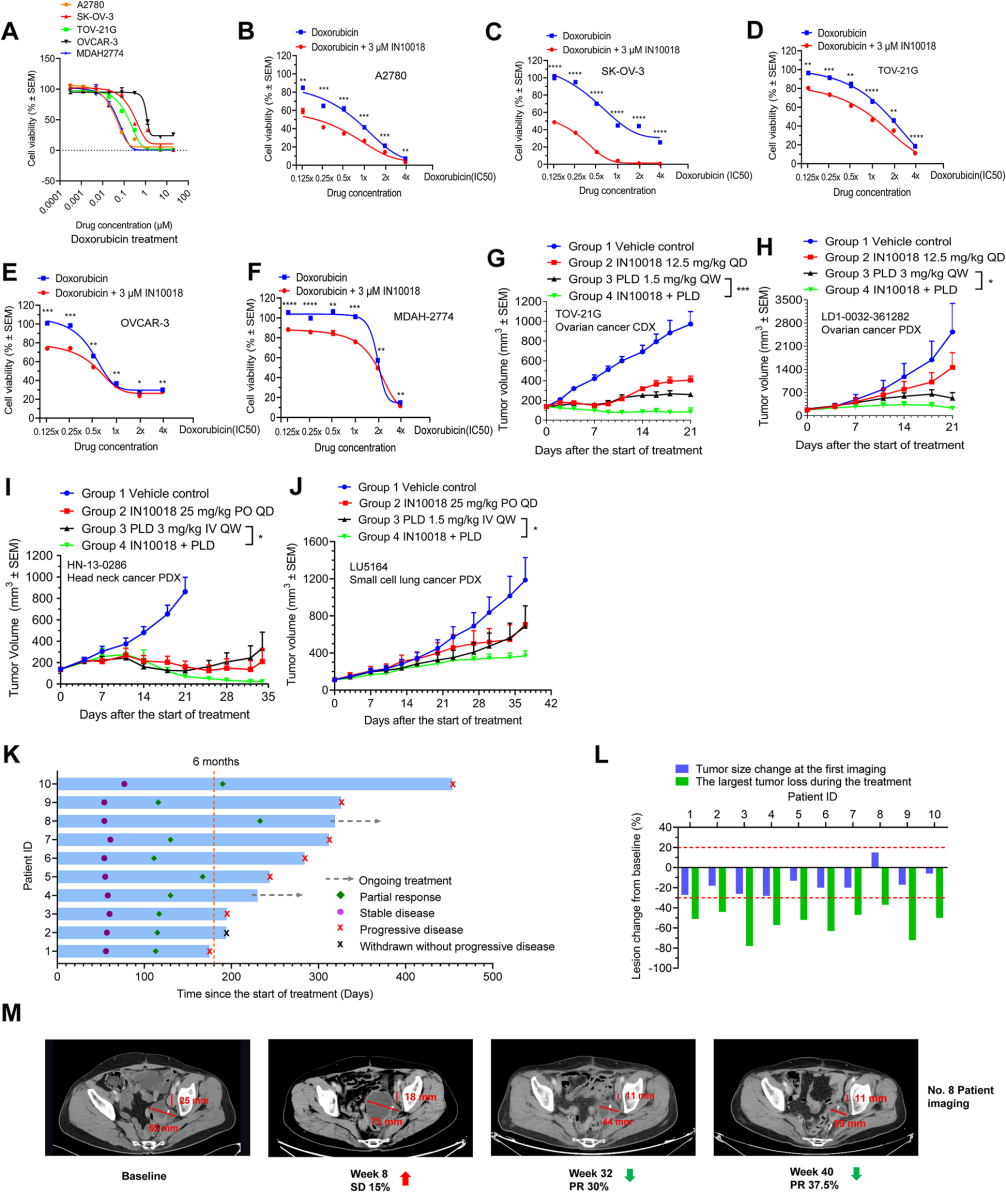
Targeting FAK synergizes with doxorubicin/PLD in the treatment of cancer in both preclinical and clinical settings. **A** Cell viability assay for doxorubicin across 5 different ovarian cancer cell lines. Cells were treated with serially diluted doxorubicin beginning with 20 μM for 72 h. The factor for each subsequent dilution is 4. (*n* = 3 per point). **B-F** Cell viability evaluation for the combination of IN10018 and doxorubicin across 5 different ovarian cancer cell lines. Each cell line was treated with 5 μM IN10018 in combination with 0.125x, 0.25x, 0.5x, 1x, 2x, or 4 × IC50 of doxorubicin. The treatment lasted 72 h. From the cell viability assay from (A-F), the CCK8 reagent was used for cell viability evaluation. (*n* = 3 per point). **G-H** Evaluation of the combined effects of IN10018 and PLD in 2 human ovarian cancer models, TOV21G and LD1-0032–361282 (n ≥ 3 per group). **I-J** The drug combination effects of IN10018 and PLD in 1 human head and neck cancer PDX model HN-13–0286 and 1 human small cell lung cancer PDX model LU5164 (n ≥ 4 per group). For animal studies from (G-J), IN10018 was dosed orally at 12.5 mg/kg or 25 mg/kg once daily. PLD was dosed at 1.5 mg/kg or 3 mg/kg by tail vein injection once weekly. **K** The swimmer plot graph for the 10 patients who showed latency responses to the combination regimen with IN10018 and PLD. The duration of responses of patients with SD, PR, and PD was assessed according to RECIST 1.1. **L** The variation of tumor sizes across the 10 patients at the first imaging and at the time showed the largest tumor loss. **M** The tumor variation of the target lesion of patient No.8 throughout the different phases of treatment with IN10018 in combination with PLD. Data represent mean ± SEM. Statistical analysis was done using the unpaired student's T-test. *P < 0.05, **P < 0.01, ***P < 0.001, and ****P < 0.0001

As for the preclinical setting, we tested the cell-killing effects after a 72 h-treatment of doxorubicin on five different ovarian cancer cell lines. Doxorubicin performed well, while IN10018 showed moderate growth inhibition to the cell lines (Fig. 2A and Fig. 1F). In drug combination tests, we administered a fixed dose of 3 μM IN10018 and combined it with serially diluted concentrations of doxorubicin for treating the selected cancer cells for 72 h. The cotreatment regimen demonstrated enhanced cell-killing effects compared to doxorubicin monotherapy (Fig. 2B-F). As doxorubicin and PLD are also clinically used for treating other cancer types [28–32], we tested the combination regimen to treat the human head and neck cancer cell line SCC9, the human breast cancer cell line MDA-MB-231, the mouse pancreatic cancer cell line KPC, the mouse colorectal cancer cell lines MC38 and CT26, the mouse non-small cell lung cancer (NSCLC) cell line KPL, and the mouse breast cancer cell line 4T1. The results across these cell types are overall similar to those from ovarian cancer cells (Supplementary Fig. 3A-H). Another clinical-stage FAK inhibitor, defactinib was also tested for validation of the combined benefit of targeting FAK and doxorubicin in vitro (Supplementary Fig. 3I-J). The drug combination containing FAK inhibition and doxorubicin had synergistic anticancer effects for all tested cell lines based on the synergy score analysis (Supplementary Fig. 5A-N).

Two ovarian cancer animal models TOV21G (CDX) and LD1-0032–361282 (PDX) were used to test the effects of drug combination (Fig. 2G-H, Supplementary Fig. 3K-L). To confirm the combination effects in other cancer types, one head and neck cancer PDX model HN-13–0286 and one small cell lung cancer PDX model LU5164 were also included in the test (Fig. 2I-J, Supplementary Fig. 3M-N). IN10018 was dosed by oral gavage once daily, and PLD was dosed by tail vein injection once weekly. The tested models demonstrated that the combination of IN10018 and PLD exerted improved antitumor effects than either monotherapy. No abnormality was observed during the dosing period.

Based on collected preclinical data, PROC was selected for a proof-of-concept study in humans (clinical trial ID: NCT05551507). Other cancer types including breast cancer, as well as head and neck cancer, will also be the subjects of forthcoming clinical tests. As of the cutoff date of 31 May 2022, A total of 50 PROC patients were enrolled and 42 of them can be evaluable for anti-tumor responses for this single-arm clinical trial (Supplementary Table 1). 90% of the patients (45/50) had 1–3 prior lines of therapy. 20% of the patients (10/50) had experienced bevacizumab and 22% of the patients (11/50) had experienced PARP1 inhibitors before. This study had a dose-confirmation part and a dose-expansion part. Dose-expansion part was performed at recommended phase 2 dose (RP2D) level to evaluate the drug responses and safety. The RP2D of IN10018 and PLD were 100 mg QD and 40 mg/m^2^ Q4W, respectively in the dose-confirmation part since no dose limited toxicity (DLT) were observed. No IN10018 related death was observed and the most frequently reported adverse effects (AEs) of IN10018 were proteinuria, decreased appetite, fatigue, and some AEs related to gastrointestinal tract, such as nausea, diarrhea, and vomiting. Most of the IN10018 related AEs were Common Terminology Criteria for Adverse Events (CTCAE) grade 1 and 2. 18% of the patients (9/50) showed grade 3 AEs. There was no grade 4 or 5 IN10018 related AEs during the study. The proteinuria was basically manageable and reversable by dosing reduction or interruption (Supplementary Table 2). The ORR was 54.8% and the disease control rate (DCR) was 88.1%. The median duration of response (DOR) was 5.8 months and the median PFS was 7.3 months.

Alongside continuous treatment with IN10018 in combination with PLD, 10 out of the patients who showed SD at the first imaging gradually exhibited partial response (PR), and their median PFS were more than 6 months (Fig. 2K-L). Intriguingly, patient No.8 exhibited 15% tumor lesion increase at the first imaging and became PR at week 32 after the start of treatment. This patient obtained sustained benefits from the treatment and showed a 37.5% tumor regression compared to the baseline at week 40 (Fig. 2M). This is akin to pseudoprogression, which is occasionally observed in response to immunotherapy [33]. We also presented the imaging evaluation for the No.4 patient who exhibited long-lasting response confronting the therapy (Supplementary Fig. 4A). Among the ten patients, two are continuing with the regimen, seven patients were withdrawn due to progressive disease (PD), and one was withdrawn upon the patient’s decision. The PR, SD, and PD individuals were evaluated based on RECIST 1.1 [34]. This observation interests us as the effectiveness differs from conventional chemotherapies but behaves more like immunotherapeutic approaches, which often have a pattern of latency followed by long-term drug responses [35]. In light of these clinical findings, we will perform experiments in preclinical settings to uncover the potential mechanism behind our observations.

### FAK inhibition propels the exposure of ICD biomarkers in the presence of doxorubicin from the cell lines of different cancer types

Our phase Ib clinical trial for the cotreatment with IN10018 and PLD exhibited long-lasting antitumor effects when comparing historical data regarding treatment with PLD alone in platinum-resistant ovarian cancer. A portion of the patients experienced latent periods before experiencing responses to the regimen [12] (Fig. 2K-M). FAK knockdown or inhibition was previously found to be synergistic with DNA damage inducers through the downregulation of NF-κB signaling [36], which then disturbs DNA repair. Therefore, the combined regimen can enhance the DNA damage burden, sequentially inducing substantial apoptosis specifically in cancer cells [37]. In this study, we found that doxorubicin can upregulate NF-κB signaling and a DNA damage biomarker, γH2AX expression in the tested cells (Supplementary Fig. 6A-C). FAK inhibition via IN10018 significantly reduced NF-κB signaling which further enhanced the over-presentation of γH2AX in the presence of doxorubicin, suggesting that IN10018 is capable of modulating the NF-κB-to-DNA damage response axis to amplify anticancer effects. However, this mechanism is not sufficient to explain the response latency and the extended treatment outcome from our clinical trial.

**Fig. 3:**
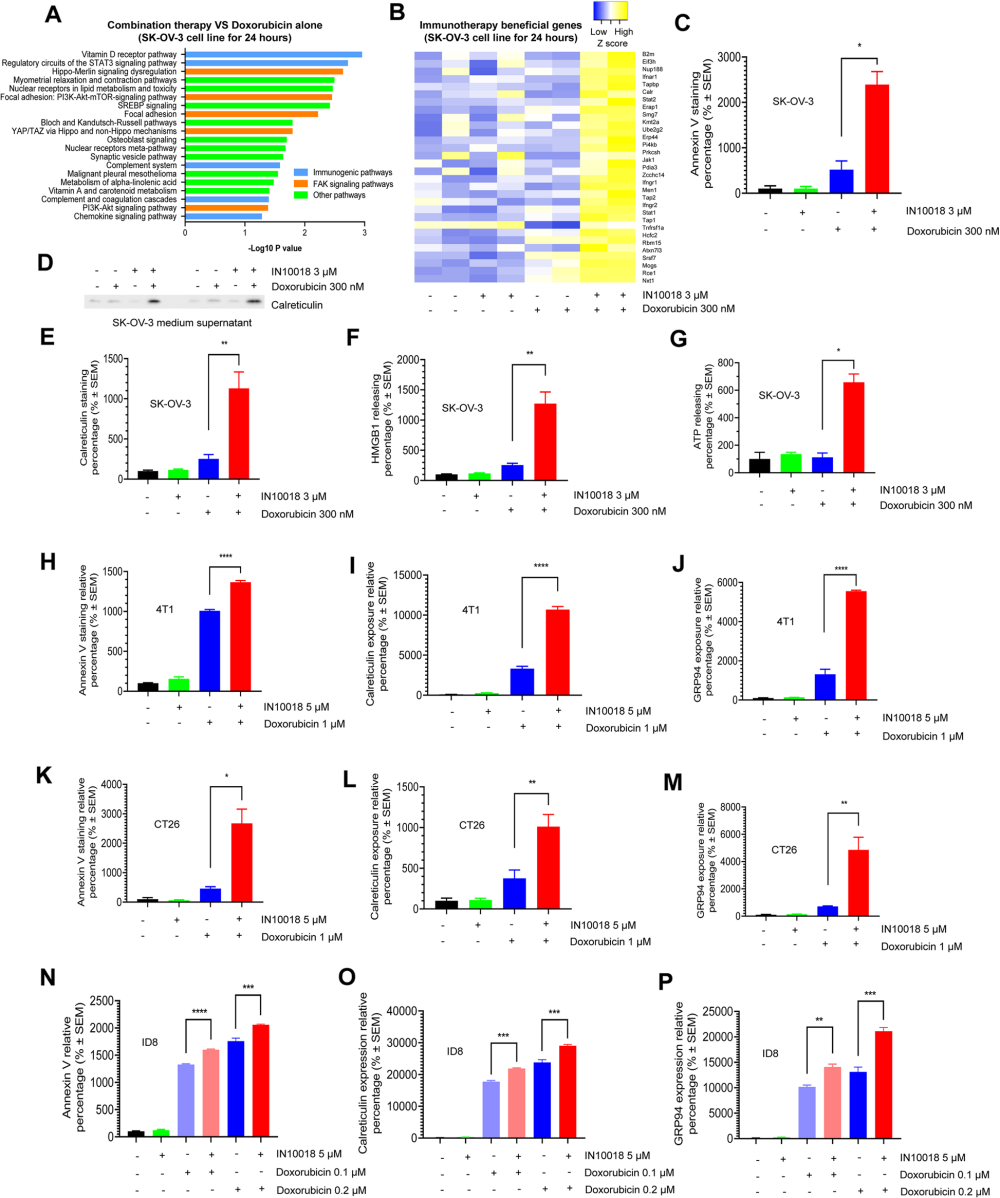
FAK inhibition propels ICD biomarker exposure in the presence of doxorubicin on the cell lines of different cancer types. **A** The most significantly altered signaling pathways upon combination treatment with IN10018 and doxorubicin. SK-OV-3 cells were treated with DMSO, 3 μM IN10018, 300 nM doxorubicin, and a combination of 3 μM IN10018 and 300 nM doxorubicin for 24 h. RNA sequencing and wiki pathway analysis were performed for the combination treatment group compared to doxorubicin monotherapy group here. The items marked in blue indicate ICD-related signaling and those marked in red indicate FAK downstream. **B** The combination of IN10018 and doxorubicin increased the expression of genes which can boost the cancer cell-killing effects CD8 positive T cells. **C** The apoptosis of SK-OV-3 cells induced by the treatment with DMSO, 3 μM IN10018, 300 nM doxorubicin, and the 2-drug combination for 48 h. (*n* = 3 per group). **D** Calreticulin releasing in the cell culture supernatant of the treated SK-OV-3 cells from (C). **E–F** Calreticulin and HMGB1 staining for treated SK-OV-3 cells from (C) (*n* = 3 per group). **G** ATP-releasing percentage of treated SK-OV-3 cells treated with indicated drugs in (C) for 24 h (*n* = 3 per group). **H-P** The annexin V/PI, calreticulin, and GRP94 staining in the mouse breast cancer cell line 4T1 (H-J), moues colorectal cancer cell line CT26 (K-M), and mouse ovarian cancer cell line ID8 (N-P) treated with different therapeutics for 48 h. The cells were stained with each antibody and analyzed using flow cytometry (*n* = 3 per point). Data represent mean ± SEM. Statistical analysis was done using the unpaired student's T-test. *P < 0.05, **P < 0.01, ***P < 0.001, and ****P < 0.0001

Transcriptome profiling was performed to determine the mechanism of the observed clinical antitumor effects. In brief, the SK-OV-3 cells were treated with DMSO, 300 nM doxorubicin, 3 μM IN10018, and a combination of IN10018 and doxorubicin for 24 h. The total mRNA was extracted for the RNA sequencing. The significantly dysregulated genes were input for Wiki pathway analysis [38]. When comparing the apparent dysregulated pathways between treatment with the combination therapy and doxorubicin alone, we found that ICD-related pathways like vitamin D receptor, STAT3 signaling, complement system, and chemokine signaling [39–42] were enriched in the combination group. FAK-driven signaling, including focal adhesion and YAP regulation, was also screened out [43] (Fig. 3A). Intriguingly, a recent report summarized the genes which exert regulatory influence on CD8-positive T cells induced cancer cell killing [44]. Our analysis demonstrated that the combined treatment resulted in overexpression of most positive regulatory genes which can boost the cancer cell killing by CD8-positive T cells compared to either monotherapy (Fig. 3B). These data, in part, highlight that ICD may be correlated with the practical outcome from the ongoing clinical trial. Interestingly, we also performed the dysregulated signaling analysis for comparison of the combination group and the IN10018 group using Wiki pathway analysis. The data showed that many of the DNA damage and cell cycle related pathways were significantly enriched in the combination group compared to FAK inhibition alone group (Supplementary Fig. 6D).

We then treated the SK-OV-3 cells with DMSO, 3 μM IN10018, 300 nM doxorubicin, and the cotreatment for 48 h. Immediately, the cells were collected for annexin V/PI staining and detection assays for the presence of DAMPs (calreticulin, HMGB1, and ATP) (Fig. 3C-G and Supplementary Fig. 7A-B). As expected, the combined treatment elicited a significant increase in cell apoptosis compared to each monotherapy (Fig. 3C). The exposure of a well-known DAMP biomarker, calreticulin can be activated multiple times by the combined therapy versus doxorubicin alone. Further, more cell release of calreticulin was induced by the drug combination compared to the monotherapies, which was confirmed by westernblot of cell culture supernatant (Fig. 3D-E). Under normal conditions, HMGB1 is restricted to inside the cell nucleus, while under intolerable pressure the protein is extruded into the cytoplasm and extracellular matrix to facilitate antigen presentation. Our data demonstrate that the combination treatment of IN10018 and doxorubicin can significantly expel HMGB1 from the cell nucleus versus the other treatment groups (Fig. 3F). The measured ATP levels from the supernatant of the cell culture also confirm that the combined regimen induced stronger ICD compared to each monotherapy (Fig. 3G). A classical ER stress biomarker, phospho EIF2α is recognized as one molecular hallmark of DAMPs [45]. Indeed, compared to the control group, we observed a clear induction of phospho EIF2α upon treatment with doxorubicin regardless of the supplementation of IN10018. The FAK inhibitor cannot further enhance its expression, indicating that phospho EIF2α may be an improper ICD target here (Supplementary Fig. 6A). To confirm that the enhanced DAMPs induction is FAK-target specific, the FAK protein level was knocked down by siRNA in SK-OV-3 cells, 24 h later followed by a 48-h treatment with doxorubicin. Calreticulin exposure was significantly improved by the cotreatment of doxorubicin and FAK siRNA compared to doxorubicin monotherapy (Supplementary Fig. 7C-D). In summary, we confirmed that targeting of FAK in combination with doxorubicin enhanced the antitumor effect. Moreover, we found that the release of ICD biomarkers was enhanced compared to doxorubicin monotherapy, providing another perspective to explain the observed outcome from the prior clinical trial.

In this study, mouse cancer cell-derived models were to be established to elucidate the efficacy and mechanism in vivo. In order to do this, we needed to confirm the induction of ICD biomarkers by the combination of FAK inhibition and doxorubicin with mouse cancer cell lines in vitro. We treated a mouse breast cancer cell line, 4T1, a mouse colorectal cancer (CRC) cell line, CT26, and another mouse ovarian cancer cell line, ID8 with DMSO, IN10018, doxorubicin, and the 2-drug combination for 48 h. The data are highly aligned with the results in human cancer cell lines. In addition, we tested the annexin V/PI staining assay for the 3 mouse cancer cell lines and overall, more apoptosis was shown by this assay after exposure to the combination regimen versus each monotherapy (Fig. 3H, K, N). The release levels of 2 DAMP biomarkers, calreticulin (Fig. 3I, L, O), and GRP94 (Fig. 3J, M, P) were analyzed using flow cytometry. Similar to the results from the SK-OV-3 cell line, the drug combination with doxorubicin and IN10018 resulted in the significantly increased release of the ICD signatures as opposed to each monotherapy in the mouse cancer cell lines.

### Targeting FAK in combination with doxorubicin significantly boosts the maturation of dendritic cells (DCs) in vitro

**Fig. 4:**
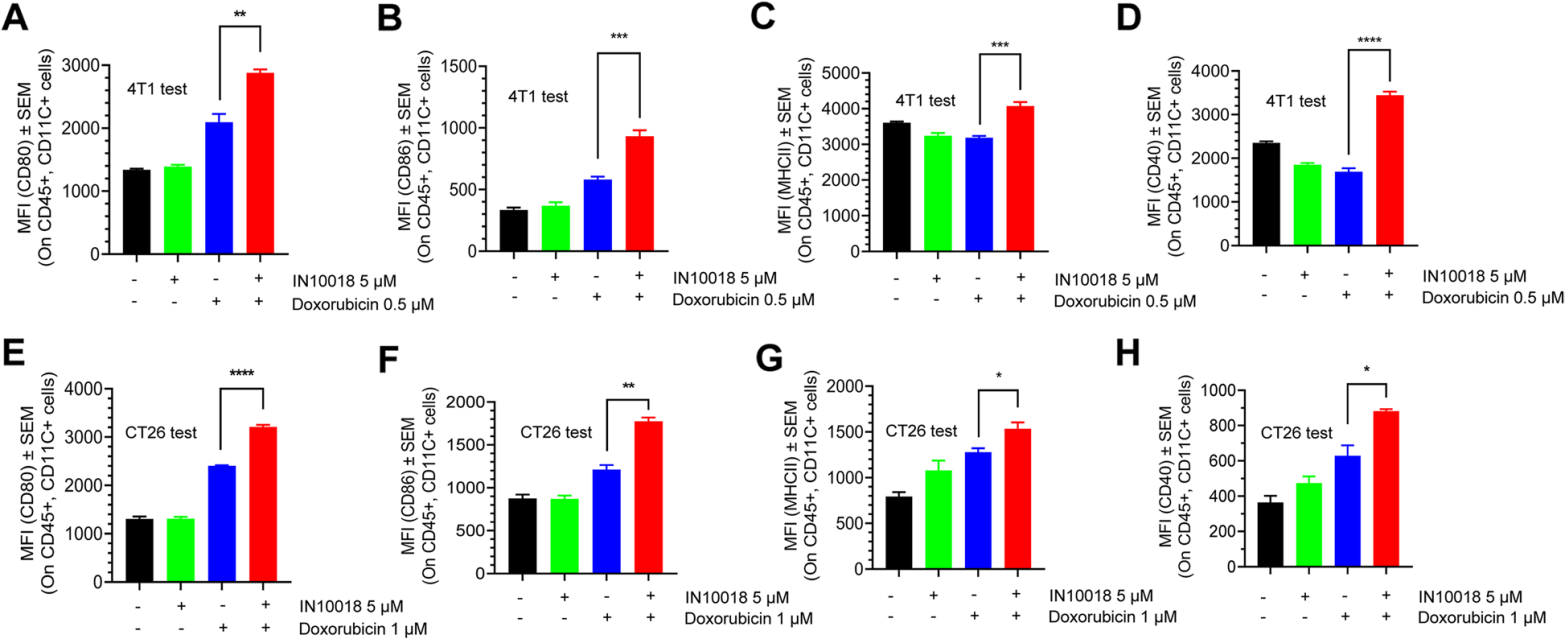
Targeting FAK in combination with doxorubicin significantly boosts the maturation of dendritic cells (DCs) in vitro. **A-D** The staining of DCs maturation biomarkers in mixed cells of treated 4T1 /CT26 and bone marrow cell pools. The 4T1 and bone marrow cells were processed as indicated in the Methods. The data were processed using GraphPad Prism 8.0 software. **E–H** The staining of DCs maturation biomarkers in mixed cells of treated CT26 and bone marrow cell pools. The data were analyzed by flow cytometry and processed with GraphPad Prism 8.0 software (n ≥ 3 per group). Data represent mean ± SEM. Statistics analysis was done using the unpaired student's T-test. *P < 0.05, **P < 0.01, ***P < 0.001, and ****P < 0.0001

The release of DAMPs induced by ICD can facilitate the recognition process of cancer-specific antigens by antigen-presenting cells (APCs) including DCs or macrophages, resulting in the maturation of APCs to present cancer antigens to naïve T cells [46]. The naïve T cells subsequently differentiate into cytotoxic T lymphocytes (CTLs) such as CD8 positive T cells to eliminate cancer cells through immune system activation. To confirm whether the ICD induced by cotreatment with FAK inhibition and doxorubicin can stimulate DCs maturation, we employed a co-culture system consisting of drug-treated 4T1 cells/CT26 cells and bone marrow-derived cells of BALB/c mice. We then examined the levels of DCs maturing biomarkers in the co-cultured mixed cells by flow cytometry. Briefly, 4T1 cells/CT26 cells were pre-cultured in presence of DMSO, 5 μM IN10018, 0.5 μM/1 μM doxorubicin, and a combination of IN10018 and doxorubicin for 72 h/48 h, respectively. The cells were collected, and the drugs were washed away with PBS. The bone marrow cell pool was prepared with femur and tibia from BALB/c mice ahead of time. GM-CSF and IL4 were supplemented into the culture to ensure sufficient innate bone marrow DCs (BMDCs) differentiation. The drug-treated 4T1 cells or CT26 cells and BMDCs were co-cultured for 96 h/72 h. The mixed cell cohorts were then stained for flow cytometric analysis with fluorescence antibodies of CD45, CD11C, CD40, MHCII, and CD86 [47]. The DCs population is observed to be CD45 positive and CD11C positive. For this cell cohort, the expression of CD40, MHCII, and CD86 which serve as biomarkers of mature DCs were significantly induced by the cotreatment of IN10018 and doxorubicin compared to the other groups in both 4T1 cells (Fig. 4A-D) and CT26 cells (Fig. 4E-H). These results indicated that the ICD induced by IN10018 in combination with doxorubicin outperformed either monotherapy in boosting DCs maturation.

### Tumor growth blockade effects of the cotreatment of FAK inhibition and PLD are related to antitumor immunity

**Fig. 5:**
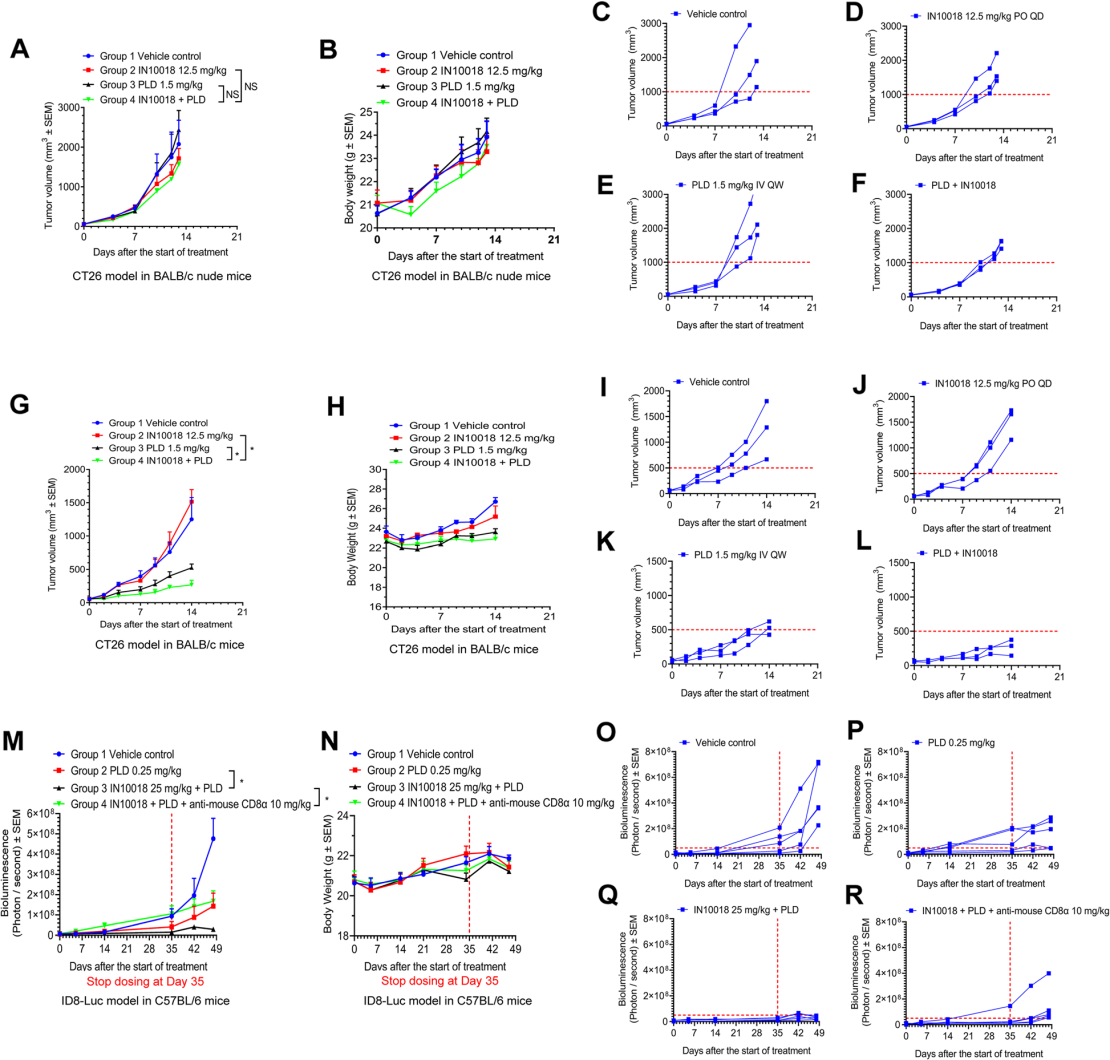
The tumor growth blockade effects by the cotreatment of FAK inhibition and PLD are related to antitumor immunity. **A-F** Treatment evaluation for the combination of IN10018 and PLD on CT26 models generated in BALB/c nude mice. IN10018 was dosed at 12.5 mg/kg through oral gavage once daily. PLD was dosed at 1.5 mg/kg by teil vein injection once weekly (*n* = 3 per group). **G-L** The treatment evaluation of the combination of IN10018 and PLD within the CT26 model generated in BALB/c mice. The treatment schedules are the same as (A-F) (*n* = 3 per group). **M-R** The peritoneal tumor sizes indicated by the bioluminescence signals and body weight records from the mouse ovarian cancer peritoneal model ID8-Luc. The model was treated for 35 days. After then, extensive observation was performed for another 2 weeks (*n* = 5 per group). Data represent mean ± SEM. Statistical analysis was done using one-way ANOVA. NS means non-significant. *P < 0.05

We have confirmed that the release of DAMPs and the maturation of DCs can be enhanced by the combination of FAK inhibition and doxorubicin in vitro. As our clinical trial employs PLD instead of doxorubicin, whether ICD-related antitumor effects can also be increased by the IN10018-PLD combination also needs to be determined. Briefly, we injected CT26 cells into immune-competent BALB/c mice as well as immune-compromised BALB/c nude mice. The experiment with BALB/c nude mice was terminated earlier due to accelerated tumor growth kinetics. After randomization, the mice in each strain were grouped into vehicle control, 12.5 mg/kg IN10018, 1.5 mg/kg PLD, and 2-drug combination, respectively. Interestingly, in the immune-compromised model, very limited antitumor effects were observed across each group (Fig. 5A-B, supplementary Fig. 8A). Individual tumor growth curves also indicated almost no tumor growth inhibition by each therapy (Fig. 5C-F). However, in the model generated with immune-competent mice, 1.5 mg/kg PLD treatment group exhibited a moderate tumor growth blockade (TGI = 60%) and the combination therapy elicited strong tumor growth inhibition (TGI = 83%) compared to the vehicle control group (Fig. 5G-H, supplementary Fig. 8B). The individual tumor growth curves also exhibited the same trend of the antitumor effects (Fig. 5I-L). The data above confirmed that the combination of IN10018 and PLD can exert immune-related cancer cell suppression in vivo.

In an animal study with mouse ovarian cancer ID8-Luc peritoneal model, PLD alone, the combination of PLD and IN10018, and the triple combination of PLD, IN10018, and anti-mouse CD8α were evaluated with their anti-tumor effects. Monotherapy with IN10018 can just exert limited anti-tumor response in this model (data not shown), so that we did not include it in this test. The animals were dosed for 35 days and kept being observed for tumor growth indicated by bioluminescence signals and body weights for another 2 weeks. In brief, this study showed that the 2-drug combination outperformed PLD monotherapy with tumor growth inhibition. While anti-mouse CD8α can rescue the drug effects from the 2-drug combination significantly. This result further suggested that anti-tumor immunity is critical to the combination benefit of PLD and IN10018 (Fig. 5M-R).

We attempted to demonstrate the anti-tumor vaccination potency of the regimen through a CT26 tumor rechallenge study. Here, doxorubicin was used for the cell assays in vitro. Briefly, we treated the CT26 cells with DMSO, 300 nM doxorubicin, and the combination of 300 nM doxorubicin and 3 μM IN10018 for 16 h in vitro. The cells were collected and instantly frozen with liquid nitrogen, then quickly thawed using 37 ℃ ultrapure water. In total, three cycles of freezing and thawing were performed to inactivate the cancer cells. The inactivated cells were then implanted into the right flanks of mice for immunization. Seven days later, living CT26 cells were implanted into the left flanks of the mice for tumor rechallenging. The tumor growth latency status represented the immunization potency of each therapy. Our results revealed that pre-inoculation with the combined regimen-treated CT26 cells had prolonged growth delay of the rechallenged CT26 tumors versus doxorubicin monotherapy (Supplementary Fig. 9G-K). These data served as a cross-validation of the ICD potency induced by the combined regimen in vivo.

### Targeting of FAK in combination with PLD induces ICD in vivo and sequentially stimulates antitumor immunity

**Fig. 6:**
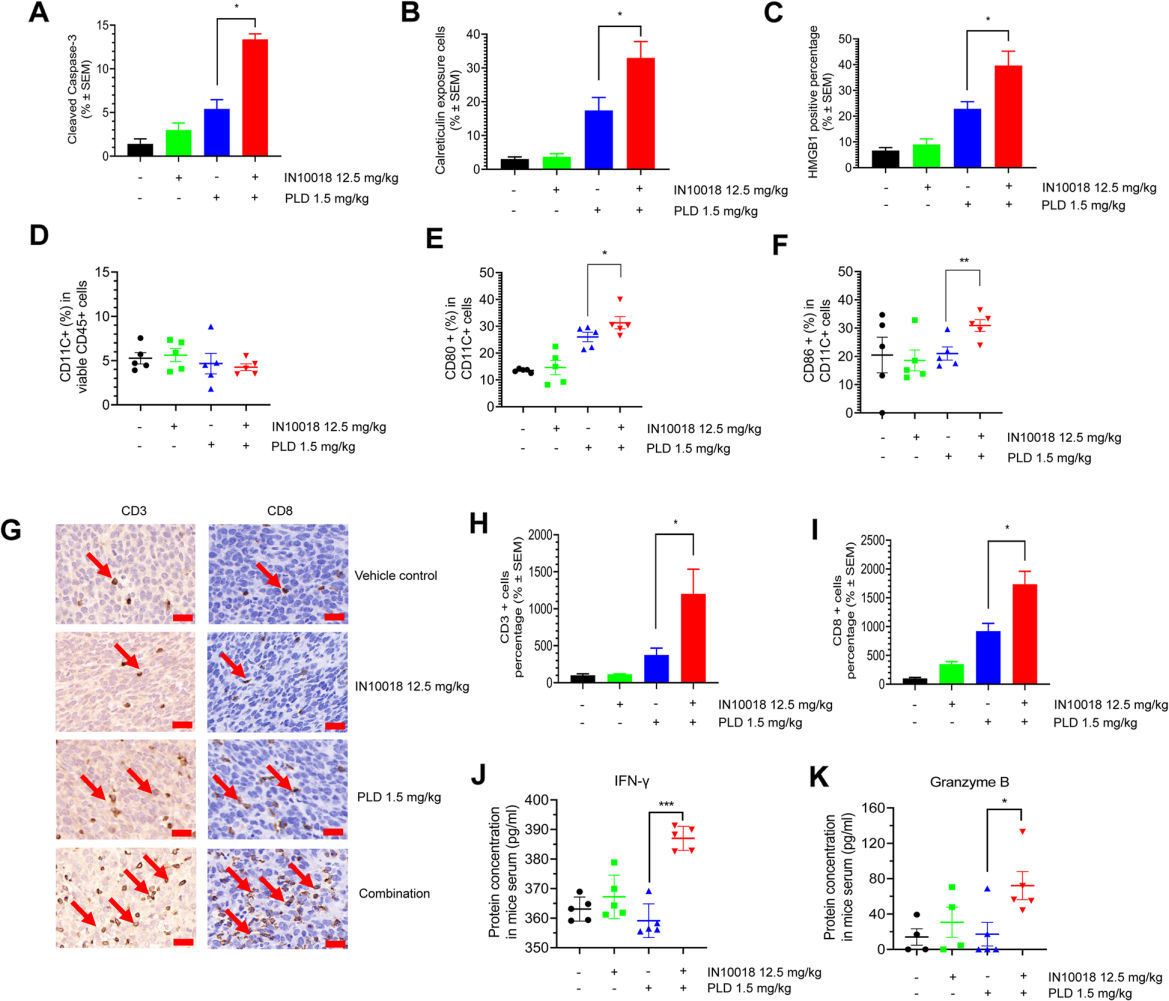
Targeting FAK in combination with PLD induces ICD in vivo and sequentially stimulates antitumor immunity. (**A**-**C**) The IHC and IF staining for tumors in the CT26 model generated in BALB/c mice from the efficacy study. Cleaved caspase-3 was stained in the tumors using IHC. Calreticulin and HMGB1 were stained using IF (*n* = 3 per group). **D-F** The DCs maturation analysis of the tumors from CT26 models treated for seven days. Flow cytometry was used for the detection (n ≥ 4 per group). **G-I** The IHC staining of CD3 and CD8 for the tumors from CT26 efficacy study in BALB/c mice. Representative staining images are shown (Scale bar = 20 μm) (G) (n ≥ 3). Arrows indicate the positive staining cells. **J-K** Serum levels of IFNγ and Granzyme B from the CT26 mice treated with drugs for seven days. The serum was extracted from whole blood for ELISA analysis of different biomarkers (n ≥ 4 per group). In all the experiments above, the positive signal percentages were assessed using ImageJ, and the data were prepared by GraphPad Prism 8.0. Data represent mean ± SEM. Statistical analysis was done using the unpaired student's T-test. *P < 0.05, **P < 0.01, and ***P < 0.001

After the CT26 animal study with immune-competent mice (Fig. 5G-H), tumor samples were collected to identify the exposure pattern of ICD molecules. Clearly, enrichment of cell apoptosis biomarkers, including cleaved caspase-3, ICD biomarkers calreticulin, and HMGB1 can be significantly exposed by the combination treatment compared to either monotherapy (Fig. 6A-C and Supplementary Fig. 9A-C), suggesting that ICD can be enhanced by IN10018 in combination with PLD in vivo. In addition, we also confirmed that FAK inhibition by IN10018 can reduce the enhanced NF-κB signaling previously induced by PLD in the testing with these tumors (Supplementary Fig. 9F).

The in vivo DCs maturation potency upon the treatment with the combination of PLD and IN10018 was evaluated with the CT26 model generated in BALB/c mice. The tested tumor-taking mice were treated with vehicle control, 12.5 mg/kg IN10018, 1.5 mg/kg PLD, and the 2-drug combination, respectively for seven days. Thereafter, the tumors were collected and processed for further flow cytometric analysis. The DCs population was determined by positive staining of CD45 and CD11C. CD80 and CD86 served as biomarkers of matured DCs. This test demonstrated that an increase in matured DCs appeared within tumors after combined therapy compared to other treatments (Fig. 6D-F and Supplementary Fig. 9L). The in vivo DCs maturation tests confirmed that FAK inhibition in combination with PLD is capable of increasing DCs maturation. Besides, we also found that the combination regimen can increase the percentage of M1 macrophages compared to the other treated groups, suggesting that the regimen is also able to regulate the macrophages other than DCs (Supplementary Fig. 9L-N). The similar trends regarding DCs maturation and immunoregulation were observed in a test with 4T1 model which was established on BALB/c mice (Supplementary Fig. 10A-I).

We conducted IHC staining for the tumor-infiltrating lymphocytes with tumor samples from our CT26 efficacy study in BALB/c mice. CD3 is a T cell-specific biomarker, and CD8 is the biomarker for cytotoxic T cells [48]. Consistent with our ICD and DCs maturation results, the data illustrated that levels of CD3- and CD8-positive cells could be increased sharply by the cotreatment of IN10018 and PLD as opposed to either monotherapy (Fig. 6G-I, Supplementary Fig. 9D-E). We also examined the serum from these animals seven days after the start of treatment. ELISA data illustrated that the combination group had significantly increased serum levels of IFNγ and Granzyme B compared to those treated with PLD alone (Fig. 6J-K). This served as another piece of evidence for enhanced antitumor immunity conferred by the doublet regimen.

### The doublet regimen containing FAK inhibition and PLD enhances the treatment outcome of ICB in mice models

FAK inhibition was shown to enhance the antitumor effects of immune checkpoint inhibition [14]. Previously, we worked with numerous syngeneic models for evaluating the inhibition efficacy of IN10018 and PD-1/PD-L1. However, our results from these experiments indicated that slight to moderate enhancement of PD-1/PD-L1 antitumor effects can be exerted by FAK inhibition (Supplementary Fig. 11A-F). To optimize the treatment outcome, we explored alternative strategies. As ICD induced by doxorubicin or PLD can be enhanced by targeting of FAK, we are interested in examining whether the cotreatment of IN10018 and PLD can further strengthen the drug effects of ICB in vivo.

We tested a triple combination therapy including 12.5 mg/kg IN10018, 1.5 mg/kg PLD, and 2.5 mg/kg anti-mouse PD-L1 in a murine syngeneic model, CT26. Notably, the triple combination regimen exhibited the best antitumor effects compared to the other tested groups (Supplementary Fig. 8C). Mice tolerated treatment well during the dosing period and the extensive observation period (Supplementary Fig. 8D). The individual tumor growth data indicated that the monotherapies or 2-drug combination therapies did not sufficiently control the tumor growth, however, the triple combination group had no apparent tumor growth throughout the study (Supplementary Fig. 8E-H). Moreover, the triplet combination of another small molecule FAK inhibitor, defactinib, PLD, and anti-mouse PD-L1 showed optimal tumor growth inhibition in the CT26 model compared to the other treated groups. This indicated that the enhanced antitumor efficacy of the ICB is related to the targeted effects of FAK (Supplementary Fig. 11I-J).

**Fig. 7:**
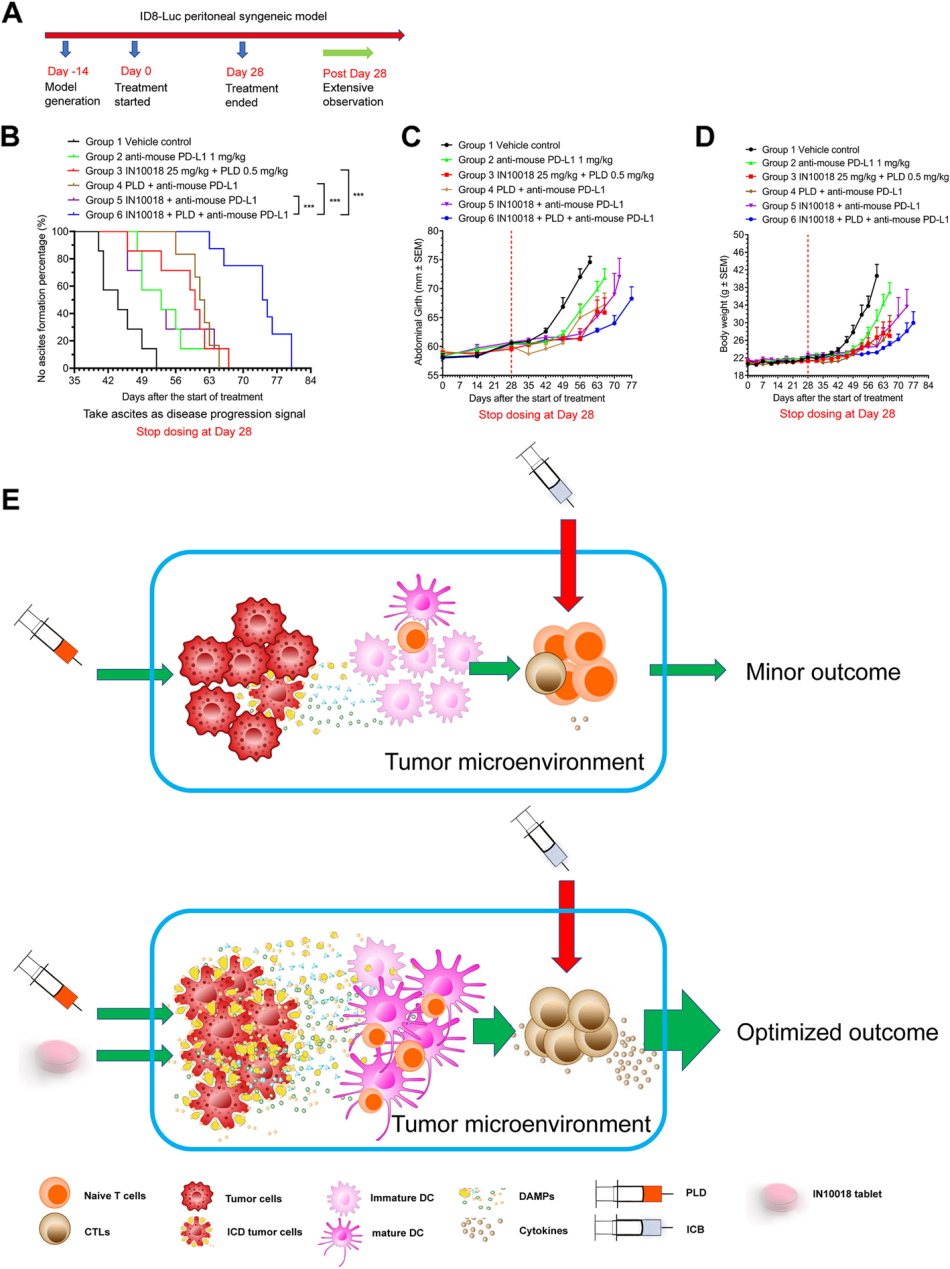
The doublet regimen of FAK inhibition and PLD enhances the treatment outcome of ICB. **A** The schedule for the ID8-Luc study. **B-D** The triple combination testing including IN10018, PLD, and anti-mouse PD-L1 in the treatment of ID8 Luc model. IN10018 were dosed through oral gavage once daily. PLD was dosed by teil vein injection once a week and anti-mouse PD-L1 was intraperitoneally administered twice a week. The dosing was stopped at day 28. The survival curves of no ascites formation rate were prepared using GraphPad Prism 8.0 software (B). The abdominal girth (C) and body weights (D) were recorded weekly. Data represent mean ± SEM. Statistical analysis was done using one-way ANOVA for the CT26 study. Log-rank test was performed for the ID8-Luc study. *P < 0.05, and ***P < 0.001. **E** Schematic model for the optimized anticancer effects of ICB boosted by the ICD from IN10018 plus PLD

Thereafter, we tested the triple combination regimen in an ovarian cancer syngeneic model, ID8-Luc. In general, the model was established with C57BL/6 mice by peritoneal injection of the cells. Fourteen days after this injection, the mice were randomized based on bioluminescence signals. When the study went to the late stage of the tumor growth, bioluminescence signals were not accurate partly due to the formation of ascites, so that we measured survival time for evaluating the disease progression instead. In brief, the treatment lasted for 28 days and mice were examined weekly until ascites formed (Fig. 7A). The survival curves determined from the time of ascites formation were drawn by each treatment. Interestingly, the triple combination group exhibited the longest ascites free time compared to the other groups (Fig. 7B). This difference in ascites formation time is statistically significant. Abdominal girth increments and body weight gain may also be used as proxy measurements for the tumor development in this model after the ascites formation. Consistently, the triple combination group exhibited the lowest body weight gain and abdominal girth increment among all treatment groups (Fig. 7C-D). In summary, the 3-drug combination of IN10018, PLD, and PD-1/PD-L1 blockade exerted outstanding antitumor effects compared to each monotherapy or 2-drug combination therapy, providing a possible clinical regimen to optimize the therapeutic response from immunotherapy. Ovarian cancer is considered to be a “cold” tumor type, which is characterized by the lack of TILs. In general, it does not respond well to ICB. Here, our data confirmed that the combination of PLD and IN10018 is capable of converting “cold” tumors to “hot” tumors, priming the tumor microenvironment for immunotherapies (Fig. 7E).

A recent study reported that targeting of FAK may provide therapeutic benefits to another immune checkpoint, TIGIT inhibition [49]. We tested the triple combination treatment of IN10018, PLD, and anti-mouse TIGIT on the CT26 syngeneic model. Similar to the tests associated with PD-1/PD-L1 blockade, the TIGIT antibody-associated triple combination also exhibited the best antitumor effects compared to other treatment groups (Supplementary Fig. 11G-H). These data suggested that the ICD induced by cotreatment of IN10018 and PLD may serve as a pan-enhancer to the blockade of immune checkpoints.

## Discussion

FAK is frequently amplified and associated with disease development in different cancer types. However, our data and other reports suggested that monotherapy inhibiting FAK may not be sufficient to obtain effective anticancer responses [17, 19]. To fully realize the impact of FAK inhibitors, combining them with other therapeutic approaches was seen as an intriguing therapeutic avenue. In this study, FAK inhibition by IN10018 can exert the highest enhancement of cancer cell killing with doxorubicin amongst different series of chemotherapeutic drugs. The liposome formulation of doxorubicin, PLD is frequently used in the clinical treatment of ovarian cancer and other cancer types through its induction of instant, severe DNA damage. FAK signaling can protect cancer cells from DNA damage-related cytotoxicity through the upregulation of NF-κB signals [36], which subsequently activate the DNA repair system [18]. Targeting FAK may enhance antitumor effects from DNA damage inducers by disrupting the DNA repair process. Based on our data and hypothesis, we confirmed that combination therapy including the FAK inhibitor IN10018 and either PLD or doxorubicin outperformed monotherapies, eliciting improved antitumor effects both in vitro and in vivo*.* In the preclinical setting, we validated that IN10018 efficiently decreased the NF-κB signaling, enhancing DNA damage in combination with doxorubicin or PLD. These data suggested that the underlying biology is similar to previous reports [36]. Finally, a proof-of-concept clinical trial for investigation of the doublet regimen was performed in PROC beginning in 2020. On the cutoff date of 31 May 2022, the response data already demonstrated outstanding therapeutic effects (overall response rate (ORR) = 54.8%, disease control rate (DCR) = 88.1%) compared to the historical data from PLD treatment alone on platinum-refractory or resistant ovarian cancer patients [50, 51]. Outside of ovarian cancer, we also performed the experiments to show the synergistic anti-cancer effects between PLD and IN10018 for pancreatic cancer, colorectal cancer, head and neck cancer, triple negative breast cancer (TNBC), small cell lung cancer (SCLC) in preclinical settings. Further, this doublet regimen will also be explored clinically in TNBC and head and neck cancer, since PLD serves as standard of care in these two cancer types.

Intriguingly, the clinical data from ovarian cancer indicated that 10 of the enrolled patients showed response latency upon treatment with the tested regimen. Most patients exhibited persistent antitumor effects, and prolonged duration of response (DOR) compared to historical data from monotherapy using PLD [12]. These data support the hypothesis that the effectiveness of this regimen may be at least partially due to antitumor immunity [52, 53]. Aligning with this hypothesis, transcriptome profiling suggested that the combination of IN10018 and doxorubicin can kill cancer cells with enhanced ICD pathways and immunogenic signatures. This indicated that ICD may ultimately be involved in the treatment outcome.

ICD is a pattern of cell death in which the surface of stressed cancer cells is damaged, causing the leakage of DAMPs to the tumor microenvironment [54]. The DAMPs stimulate APCs presenting tumor-specific antigens to innate T cells, activating more CTLs for long-term antitumor effects [55]. PLD and its active ingredient, doxorubicin are certainly ICD inducers. The former has already been evaluated on PD-1/PD-L1 blockade and exhibited good antitumor effects in preclinical and clinical settings [9, 56]. Targeting of FAK was also demonstrated effective in increasing antitumor immunity and exerting increased efficacy to ICB [14, 57]. In alignment with emerging evidence, we confirmed that heightened levels of DAMPs could be released by cancer cells under treatment with IN10018 in combination with either doxorubicin or PLD as opposed to monotherapies. Furthermore, the increased ICD is sufficient to improve the maturation of DCs and the infiltration of CTLs in tumors. The animal models generated in immune-proficient versus immune-deficient mice responded to the cotreatment of IN10018 and PLD much better, mechanistically conferring a clear interpretation of the clinical outcome.

FAK operates as a multi-featured chaperone in regulating critical processes during cancer development, including antitumor immunity, cancer cell stemness, epithelial-mesenchymal transition, and the tumor microenvironment [19, 58]. Concomitant targeting of FAK and immune checkpoints is known to exert synergistic antitumor effects by activating antitumor immunity [57, 59] and remodeling the immunosuppressive microenvironment [14]. These characteristics of FAK led us to evaluate the drug combination efficacy including IN10018 and PD-1/PD-L1 inhibition. Unexpectedly, multiple animal studies have revealed that outcomes of the combined treatment are severely limited. Another FAK inhibitor, defactinib has been employed in combination with the PD-1 antibody pembrolizumab in a clinical trial beginning in 2016 targeting advanced solid malignancies. This project was closed without data publication, yet recently, a study testing a triple combination of defactinib, gemcitabine, and pembrolizumab has published preliminary clinical data [60]. The emerging evidence suggested that targeting FAK alone may not be sufficient to optimize the antitumor efficacy of ICB. Coincidentally, the cotreatment of PLD with PD-1 blockade in ovarian cancer patients has already been assessed clinically. Although the efficacy is much more refined compared to PD-1 inhibition or PLD alone, the ORR is approximately 25%, indicating that this regimen still has room to be optimized [24].

A recent report indicated that FAK inhibition could eliminate cancer-associated fibroblasts (CAFs) which are responsible for the suppression of antitumor immunity, thereby increasing the antitumor effect of radiotherapy. This report further tested a triplet combination of FAK inhibitor, radiotherapy, and PD-1 antibody and uncovered that antitumor effects were enhanced significantly compared to the other tested treatments [61]. In our setting, as ICD induction can be significantly enhanced by IN10018 and either doxorubicin or PLD, we carefully tested triplet therapy including FAK inhibition, PLD, and ICB to see if it was capable of further enhancing the antitumor effects safely and effectively. The in vivo experiments utilizing PD-L1 or TIGIT blockade with CT26 and ID8-Luc animal models confirmed our hypothesis as the triplet regimen exhibited prolonged and refined antitumor effects. The encouraging results from preclinical tests warrant a further validation clinical trial for the triple combination of IN10018, PLD, and ICB.

In summary, we experimentally confirmed that FAK inhibition by IN10018 can enhance the ICD induced by PLD, further amplifying the antitumor effects of ICB. We expect to provide a safe and effective triplet regimen for the clinical treatment of ovarian cancer and other cancer types.

## Methods

### Cell lines and reagents

The cancer cell lines A2780, SK-OV-3, PA-1, TOV21G, TOV112D, OVCAR3, MDA-MB-231, MC38, 4T1, CT26, SCC-9, B16-F10, and MDAH-2774 were acquired from ATCC. The Kuramochi, A1847, SNU251, COV362, ID8, and Pan02 cell lines were from Cobier. The ID8-Luc cell line was from Pharmalegacy. The mouse KPC cell line was purchased from Modelorg. The mouse KPL cell line was a gift from Ji et al. [62]. All cells were maintained in RPMI1640 or DMEM (Basalmedia) supplemented with 10% fetal bovine serum (FBS) (Gibco). IN10018 was provided by InxMed. The chemotherapeutic drugs used for screening were purchased from Medchemexpress. Doxorubicin and defactinib were purchased from SuperLan-chem, and PLD for animal studies was from Jingyuan. FAK siRNA was synthesized by Genepharma, and the sequence is listed in the Supplementary Table 3. The antibodies used in the study are listed in Supplementary Table 4.

### Anti-mouse TIGIT production

The anti-mouse TIGIT (10A7 clone) for in vivo testing was generated in house. In brief, the CHO cells were transiently transfected to express the anti-mouse TIGIT. The sequence of generated antibody was from patent US20090258013A1 and is shown in Supplementary Table 3.

### Cell viability assay

Cell viability was evaluated with CCK8 reagent (Cellorlab). Briefly, cells were dispensed into 96 well plates. After the cells attached to the well surface, the agents were administered to each well. The cells were treated for 72 h. 10 μL of CCK8 reagent was then added to each well. The cells were incubated with CCK8 for another 2 to 4 h at 37℃. Finally, the signal at 450 nm was recorded using a plate reader (Molecular devices). The cell viability fraction based on signal readout was analyzed and calculated by GraphPad Prism 8.0.

### Chemotherapeutic drug screening

Human ovarian cancer cell lines A2780, SK-OV-3, the mouse colorectal cancer cell line CT26, and the mouse pancreatic cancer cell line KPC were used in drug screening. The drug pool contained 21 conventional chemotherapeutic drugs. In brief, the drugs were diluted with DMSO-containing medium to different indicated concentrations, then added to each well of the 96 well plates. The cells were added to the wells with or without 3 μM IN10018 and maintained in a cell incubator for 72 h. The cell plates were then moved out of the incubator, and the CCK8 reagent was used to evaluate cell viability. Finally, the log IC50 values of chemotherapy alone (x-axis) or combined with IN10018 (y-axis) were placed in a graph to illustrate the cell-killing preference of different regimens.

### Immunofluorescence assay

4% paraformaldehyde (Beyotime) was used to fix the cells. Cells were then incubated with 0.1% Triton X (Sigma-Aldrich) for permeation. 3% BSA (Sangon) was used for 1 h to block the cells at RT. The primary antibody was added to the cells for 2 h at RT. The cells were then supplemented with Hoechst33342 (Cell Signaling Technology) and a secondary antibody with fluorescence (Cell Signaling Technology) for 1 h at RT. The cell-containing slides were sealed using the fluorescence mounting medium (Beyotime). Images were taken using an Olympus U-HGLGPS microscope. Statistical analyses were performed using ImageJ and GraphPad Prism 8.0.

### Flow cytometry

Cells were placed into 6 well plates and treated with test articles for a determined length of time. The cells were then collected in a tube for flow cytometry. Fluorescence-labeled antibodies were added to the cells for 30 min. Staining intensities were recorded and analyzed by FACSCanto II flow cytometry (BD Bioscience). The results were analyzed using Flowjo 10.0.

### Westernblot

Protein samples were collected from cell culture or tumor tissues using RIPA lysis buffer (Rockland). A BCA kit from Thermos Fisher Scientific was used for the quantitation of protein level. The protein lysis buffer was mixed with 4 × laemmli blue loading buffer (Bio-Rad) and boiled at 95℃ for 15 min. A 12% SDS-PAGE gel was used for resolving the protein by molecular weights. A nitrocellulose membrane (Bio-Rad) was used for protein transfer. After transferring, the samples were incubated with primary antibodies overnight at 4℃. On the second day, the samples were incubated with HRP-labeled secondary antibodies for 1 h at RT. A ChemiDoc MP (Bio-Rad) was used for membrane exposure after excitation with the ECL reagent (Epizyme biomedical).

### Apoptosis assay

After drug treatment, cells were fixed and stained with annexin V/propidium iodide (PI) kit (Beyotime). The experiment was conducted as described by the protocol. After staining, the cells were transferred for flow cytometry to detect apoptosis. Annexin V positive/PI negative cohort represents early apoptotic cells, and the annexin V positive/PI positive cohort represents late apoptotic cells.

### ATP detection assay

The extracellular ATP was evaluated using an ATP detection kit from Beyotime. A standard curve of ATP was drawn with different ATP concentrations. The cell culture medium was collected and used as samples. The ATP detection reagent was added to the samples, and the luminescence readout was recorded using a plate reader (Molecular devices).

### siRNA transfection

Cells were transfected with siRNAs using lipofectamine 3000 reagent (Thermo Fisher Scientific). Serum-free medium Opti-MEM (Gibco) was used to prepare siRNA-containing buffer and lipofectamine-containing buffer. The two fractions were mixed and incubated together for 20 min at RT. The mixture was then added dropwise to the cell culture. The biomarkers and phenotype changes were observed after transfection.

### In vitro test for DCs maturation

Bone marrow cells of 3 BALB/c mice were collected from the bone marrow of the tibia and femur. The cells were aspirated to obtain a cell suspension for further analysis. 10 ng/μL IL4 (Biolegend) and 10 ng/μL GM-CSF (Abcam) were added to the bone marrow-derived cells to promote the initial differentiation of DCs. The 4T1/CT26 cancer cells were treated with DMSO, 5 μM IN10018, 0.5 μM doxorubicin, and the drug combination for 72/48 h. Then, the treated 4T1/CT26 cells were washed with PBS three times and co-cultured with bone marrow-derived cells for extra 96/72 h. The cells were harvested for staining of the fluorescence antibodies of CD45, CD11C, CD80, CD86, CD40, and MHCII and were analyzed by flow cytometry to uncover the maturation rate of DCs.

### RNA-seq

Total RNA was extracted following the protocol of the RNA iso plus reagent (Takara). RNA integrity values were evaluated using an RNA 6000 nano kit on a 2100 bioanalyzer (Agilent). The cDNA libraries were prepared using the NEBNext Ultra Directional RNA Library Prep kit for Illumina (New England Biolabs). An Illumina Hiseq 2500 sequencer was selected for sequencing. Raw data were checked with FASTQC software and then processed with TopHat and Cuffdiff packages [63, 64]. Wiki pathway analysis was used to filter out the most significantly disrupted signaling by the treatment. The Heatmapper software package [65] was used to draw a heatmap of immunotherapy-related genes.

### Animal study

6- to 8-week-old mice were selected for model generation. In brief, for the cell line-derived xenograft (CDX) models, A2780 (1 × 10^7^), PA-1 (1 × 10^7^), TOV21G (5 × 10^6^), OVCAR3 (1 × 10^7^), TOV112D (2 × 10^6^), and SK-OV-3 (1 × 10^6^) were injected into the right flank of each mouse for model establishment. For the mouse syngeneic models, 3 × 10^5^ CT26 cells were injected into the right flank of each BALB/c mouse. 2 × 10^5^ 4T1 cells were used for model generation on BALB/c mice. 1 × 10^6^ of Pan02 cells and B16-F10 cells were inoculated into the right flank of each C57BL/6 mouse, respectively to generate mice models. 5 × 10^6^ ID8-Luc cells were injected into the peritoneal cavity of each C57BL/6 mouse to generate an ascites formation model. For the patient-derived xenograft (PDX) models, the seed tumors were sliced into pieces (around 30 mm^3^ for each) and implanted into the right flanks of the mice. The PDX models were established on nude mice. The solvent for IN10018 was 0.5% Natrosol 250 HX in distilled water. PLD, anti-mouse PD1, anti-mouse PD-L1, and anti-mouse TIGIT were prepared using 0.9% saline. The tumor volumes and body weight changes were recorded twice a week. Tumor volumes were measured by caliper and estimated with a formula of 0.5 × long diameter x short diameter^2^. Dosing began when the tumor volume reached 50–300 mm^3^ in the subcutaneous models. For the ID8-Luc peritoneal model, mice were randomized and recorded based on bioluminescence signals at day 14 after inoculation, abdominal girths were recorded for checking the formation of ascites. The CT26 tumors from BALB/c mice were collected for immunohistochemistry and westernblot. Serum from the mice was collected on day 7 for ELISA testing of chemokines, including IFN-γ and Granzyme B.

### Animal study approval

All animal studies were performed according to the protocols approved by the ethics committee of Shanghai Jiao Tong University School of Medicine (SJTU-SM) and conducted following the AAALAC guidelines. In detail, animal experiments with A2780, PA-1, TOV21G, OVCAR3, TOV112D and SK-OV-3, TOV21G, CT26, and 4T1 models were approved by the Institutional Animal Care and Use Committee (IACUC) of Boehringer-Ingelheim, Nanjing Yunqiao and Shanghai Sixin. The studies with PDX models of LD2-0032–200651, LD2-0032–200775, and LD1-0032–361282 were approved by the IACUC of Shanghai Lide. The study using the PDX model of HN-13–0286 was approved by the IACUC of Shanghai WuXi AppTec. The study using the PDX model of LU5164 was approved by the IACUC of Crownbio. The study using mouse syngeneic model B16-F10 was approved by the IACUC of Shanghai Modelorg. The study using mouse syngeneic model ID8-Luc was approved by the IACUC of Shanghai Pharmalegacy.

### Flow cytometry tests for the in vivo study

6- to 8-week-old BALB/c mice were selected for testing. The CT26/4T1 cells were prepared and inoculated to the right flanks of the mice. After tumor establishment, the animals were randomized into subgroups with different treatments. For the CT26 study, on day 7 after treatment startup, tumor samples were excised for DCs maturation analysis. The tumors were lysed into cell suspensions using a tumor dissociation kit (Miltenyi). The cell suspensions were stained using CD45, CD11B, CD11C, CD80, and CD86 fluorescence antibodies and was analyzed by flow cytometry to uncover the maturation rate of DCs and staining of macrophages. For the 4T1 study, on day 13 post the start of treatment the tumors were harvested and processed into cell suspensions. Then, CD45, CD3, CD4, CD8, CD25, FOXP3, CD11C, and CD80 of the samples were stained with fluorescence antibodies for detecting CD3 + T cells, CD4 + T cells, CD8 + T cells, Treg cells, DCs and DCs maturation, respectively.

### ELISA assay

The serum was separated from the whole blood of the mice. The serum IFN-γ and Granzyme B levels were measured using ELISA kits following manufacturer instructions. IFN-γ ELISA kit was purchased from Beyotime. The Granzyme B ELISA kit was supplied by Boster.

### CT26 rechallenging study

6- to 8-week-old BALB/c mice were selected for the rechallenging study. The CT26 cells were treated with DMSO, 300 nM doxorubicin, and the combination of 300 nM doxorubicin and 3 μM IN10018 for 16 h. Cells were then harvested and exposed to three cycles of freezing and thawing using liquid nitrogen and a 37℃ water bath. The processed cells were seeded into the right flanks of the mice. Seven days later, live CT26 cells were injected into the left flanks of the mice. Tumor sizes and taking rates were recorded for the evaluation of immunization potency.

### Pathological analysis

The tumor tissues of this study were harvested, fixed with 4% paraformaldehyde (Sigma-Aldrich), and prepared into formalin-fixed paraffin-embedded (FFPE) blocks. The pathology slides were stained with primary and secondary antibodies for Immunohistochemistry and immunofluorescence. to mark the region of interest. KF-PRO-120 scanner (KFBIO) was used to scan the pathology slides. Image J was used for the analysis of the scanned images.

### Clinical trial

The clinical trial was approved by Cancer Hospital Chinese Academy of Medical Sciences, Beijing, China. Written informed consent was obtained from all patients. The clinical trial ID of the combination treatment with PLD and IN10018 for PROC is NCT05551507. We updated the clinical readout here based on data on the cutoff date of 31 May 2022. In this study, we selected and analyzed the data from 10 patients who demonstrated SD at first imaging and gradually became PR throughout the trial. The protocol number for this clinical trial is IN10018-006. For the clinical trial, tumor sizes were assessed through computed tomography (CT) scanning or magnetic resonance imaging (MRI). The SD, PR, and PD were evaluated based on RECIST1.1 [34]. The graphs of tumor size variation, swimmer plot, and imagining data were prepared to present the treatment outcome. In the clinical trial, PLD was purchased from CSPC and IN10018 was supplied by InxMed.

### TCGA, MCLP, and CTRP data analysis

The copy number values of the *PTK2* gene across different cancer types, *PTK2* mRNA and protein levels, overall survival, and progression-free survival data from ATCC data collection were obtained from the cBioPortal website. The survival analysis for patients with “copy number values > 7” and “no copy number variance” was performed in GraphPad Prism 8.0. The expression levels of phospho FAK Y397 across different cancer cell lines were downloaded from the MCLP database. The cell viability data from drug screening with PF-573228 were downloaded from the CTRP database. These datasets were prepared and displayed by GraphPad Prism 8.0.

### Statistics

Each data point displayed is represented as Means ± SEM. Correlations between different parameters were analyzed using a slope coefficient test. The synergy effects for in vitro assays were represented by synergy scores evaluated through synergy finder 2.0 [66]. The statistical significance of differences between the tested groups was determined using an unpaired two-tailed Student’s *t-*test or one sample *t-*test. One-way ANOVA with Dunnett’s method was tested for comparisons of multiple groups. Log-rank analysis was performed to evaluate the significance between survival curves. Throughout the whole study, the statistical analyses were performed with GraphPad Prism 8.0 software. *P* values less than 0.05 were considered to be significant, *P < 0.05, **P < 0.01, ***P < 0.001, ****P < 0.0001.

### Supplementary Information

**Additional file 1:** **Supplementary Figure 1.** Phospho FAK Y397 levels, sensitivity to FAK inhibitor PF-573228 of different cancer cell lines, and drug screening results from CT26 and KPC cell lines. **Supplementary Figure 2.** IHC staining for FAK target in different ovarian cancer tumor tissues. **Supplementary Figure 3. **The antitumor benefits of the combination of FAK inhibition and doxorubicin/PLD. **Supplementary Figure 4. **The tumor images from No.4 patient. **Supplementary Figure 5.  **The synergistic analysis for the combination treatment with doxorubicin and FAK inhibitors in vitro. **Supplementary Figure 6. **The regulation of NF-κB and DNA damage signaling in part serves as the mechanism behind the drug combination effects of IN10018 and doxorubicin. **Supplementary Figure 7.** The representative images for the immunofluorescence staining of cancer cells treated with the combination of FAK inhibition/knockdown and doxorubicin. **Supplementary Figure 8.** The tumor images from the animal studies of CT26 model and the tumor growth inhibition for triple combination of PLD, IN10018, and PD-L1 blockade. **Supplementary Figure 9. **The IHC, IF staining, and westernblot for the tumors from the combination test with IN10018 and PLD on CT26 syngeneic model and the CT26 rechallenging tests. **Supplementary Figure 10. **The animal study data for the combination treatment of FAK inhibition and PLD in the treatment of 4T1 model. **Supplementary Figure 11. **The animal study data for the combination treatment of FAK inhibition, PLD, and immune checkpoint inhibition. **Supplementary Table 1. **The characteristics, treatments, and responses of the efficacy-evaluable patients in the clinical trial with dual regimen of PLD and IN10018 in the treatment of PROC. **Supplementary Table 2. **The AEs summary for the clinical trial. **Supplementary Table 3. **The sequences of FAK siRNA and anti-mouse TIGIT used in the study. **Supplementary Table 4. **The antibodies used in the study.

## Data Availability

The RNA-Seq data have been deposited in the NCBI’s Gene Expression Omnibus (GEO) database under the accession code GSE233112. The TCGA data used in the manuscript can be downloaded from cBioPortal (https://www.cbioportal.org/). All the other data generated or analyzed during this study are included either in this article or in additional files.
