# Correction: Targeting of focal adhesion kinase enhances the immunogenic cell death of PEGylated liposome doxorubicin to optimize therapeutic responses of immune checkpoint blockade

**DOI:** 10.1186/s13046-024-03033-8

**Published:** 2024-04-16

**Authors:** Baoyuan Zhang, Ning Li, Jiaming Gao, Yuxi Zhao, Jun Jiang, Shuang Xie, Cuiping Zhang, Qingyu Zhang, Leo Liu, Zaiqi Wang, Dongmei Ji, Lingying Wu, Ruibao Ren

**Affiliations:** 1 State Key Laboratory for Medical Genomics, Collaborative Innovation Center of Hematology, Shanghai Institute of HematologyNational Research Center for Translational Medicine Ruijin Hospital, Shanghai Jiao Tong University School of Medicine, Shanghai, China; 2 Department of Gynecologic Oncology, National Cancer Center, National Clinical Research Center for Cancer/Cancer Hospital, Chinses Academy of Medical Sciences and Peking Union Medical College, Beijing, China; 3 Department of Medical Oncology, Fudan University Shanghai Cancer Center, Shanghai, China; 4 International Center for Aging and Cancer, Hainan Medical University, Hainan Province, Haikou, China; 5 InxMed (Shanghai) Co., Ltd, Beijing, China; 6 Department of Pathology, Yantai Afliated Hospital of Binzhou Medical University, Yantai, Shandong China; 7 Laboratory of Obstetrics and Gynecology, Afliated Hospital of Guangdong Medical University, Zhanjiang, Guangdong China

**Correction:** ***J Exp Clin Cancer Res*** **43, 51 (2024)**

10.1186/s13046-024-02974-4

Following publication of the original article [1], Baoyuan Zhang, Ning Li, Jiaming Gao were not captured as co-first authors and equal contributors. This was not declared in the accepted manuscript and the authors failed to correct this during proofing.

The correction does not affect the overall result or conclusion of the article. The original article has been corrected.
