# Supplementary material for: Targeting of focal adhesion kinase enhances the immunogenic cell death of PEGylated liposome doxorubicin to optimize therapeutic responses of immune checkpoint blockade
Source: J Exp Clin Cancer Res. 2024 Feb 19;43:51. doi: 10.1186/s13046-024-02974-4 (PMC10875809; doi:10.1186/s13046-024-02974-4)
Supplement: Supplementary file 1 — Additional file 1: Supplementary Figure 1. Phospho FAK Y397 levels, sensitivity to FAK inhibitor PF-573228 of different cancer cell lines, and drug screening results from CT26 and KPC cell lines. Supplementary Figure 2. IHC staining for FAK target in different ovarian cancer tumor tissues. Supplementary Figure 3. The antitumor benefits of the combination of FAK inhibition and doxorubicin/PLD. Supplementary Figure 4. The tumor images from No.4 patient. Supplementary Figure 5. The synergistic analysis for the combination treatment with doxorubicin and FAK inhibitors in vitro. Supplementary Figure 6. The regulation of NF-κB and DNA damage signaling in part serves as the mechanism behind the drug combination effects of IN10018 and doxorubicin. Supplementary Figure 7. The representative images for the immunofluorescence staining of cancer cells treated with the combination of FAK inhibition/knockdown and doxorubicin. Supplementary Figure 8. The tumor images from the animal studies of CT26 model and the tumor growth inhibition for triple combination of PLD, IN10018, and PD-L1 blockade. Supplementary Figure 9. The IHC, IF staining, and westernblot for the tumors from the combination test with IN10018 and PLD on CT26 syngeneic model and the CT26 rechallenging tests. Supplementary Figure 10. The animal study data for the combination treatment of FAK inhibition and PLD in the treatment of 4T1 model. Supplementary Figure 11. The animal study data for the combination treatment of FAK inhibition, PLD, and immune checkpoint inhibition. Supplementary Table 1. The characteristics, treatments, and responses of the efficacy-evaluable patients in the clinical trial with dual regimen of PLD and IN10018 in the treatment of PROC. Supplementary Table 2. The AEs summary for the clinical trial. Supplementary Table 3. The sequences of FAK siRNA and anti-mouse TIGIT used in the study. Supplementary Table 4. The antibodies used in the study. [file 13046_2024_2974_MOESM1_ESM.docx]

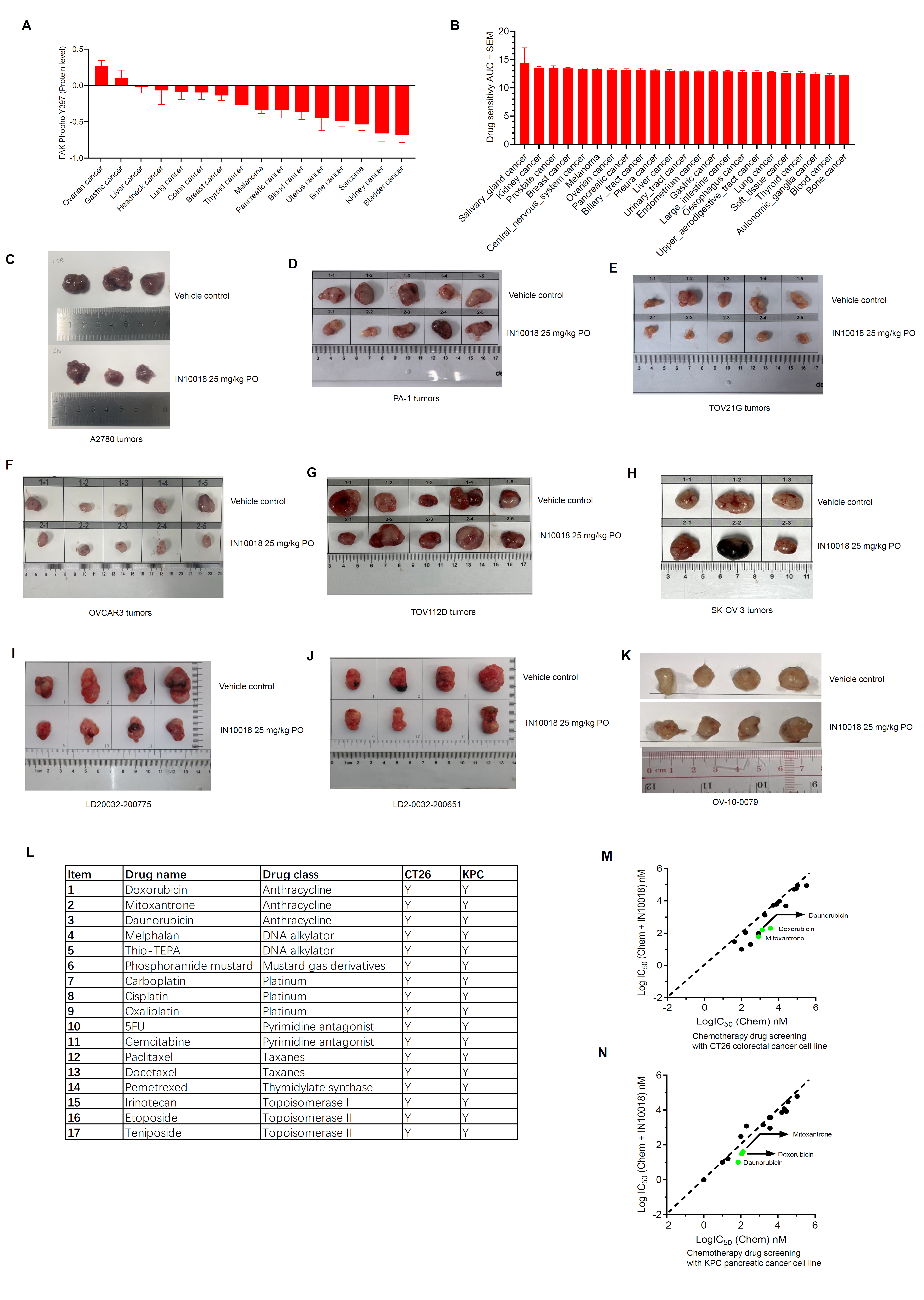


**Supplementary Figure 1:** Phospho FAK Y397 levels, sensitivity to FAK inhibitor PF-573228 of different cancer cell lines, and drug screening results from CT26 and KPC cell lines.

**(A)** Phospho FAK Y397 expression levels of different cancer cell lines from MCLP project. **(B)** The anti-cancer responses to FAK inhibitor PF-573228 of different cancer cell lines from the cancer therapeutics response portal. The drug sensitivity was shown by drug sensitivity AUC here. **(C-K)** The tumor images upon the completion of the animal studies for Fig. 1H. **(L)** The drug information of the screening assay with CT26 cells. **(M)** The results of the chemotherapeutic screening with CT26 cells. **(N)** The results of the chemotherapeutic screening with KPC cells.


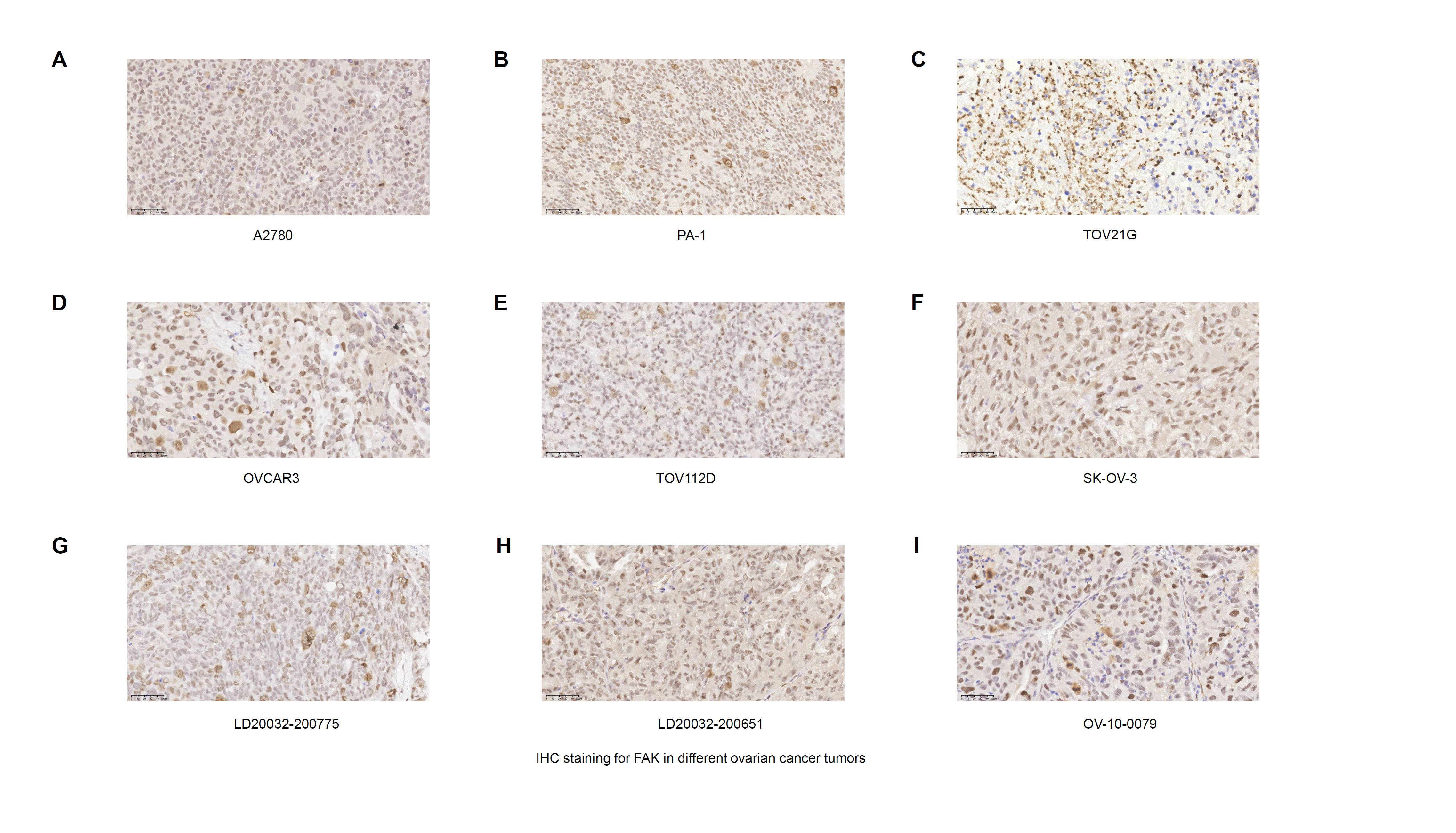


**Supplementary Figure 2:** IHC staining for FAK target in different ovarian cancer tumor tissues.

**(A-I)** The IHC staining images for FAK targets of the ovarian cancer tumor tissues from Fig. 1H.


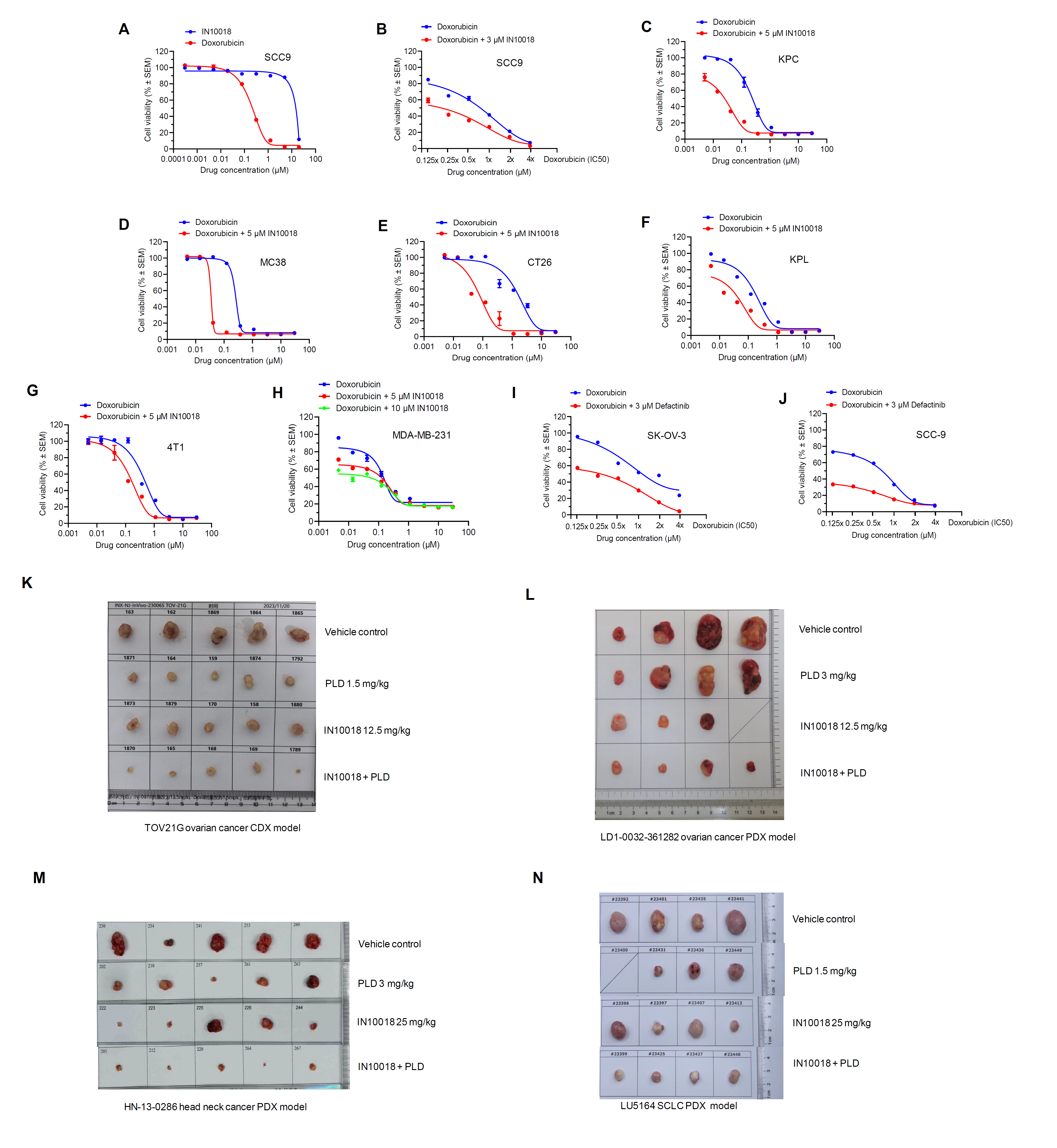


**Supplementary Figure 3:** The antitumor benefits of the combination of FAK inhibition and doxorubicin/PLD.

**(A-H)** The cell viability assay for testing IN10018 in combination with doxorubicin with human head and neck cancer cell line SCC-9, mouse pancreatic cancer cell line KPC, mouse colorectal cancer cell line MC38 and CT26, mouse non-small cell lung cancer cell line KPL, mouse breast cancer cell line 4T1 and human breast cancer cell line MDA-MB-231. The cells were treated with doxorubicin in combination with 3 μM or 5 μM IN10018 (n = 3 per point). **(I-J)** The cell viability tests for the combination of defactinib and doxorubicin on SK-OV-3 and SCC-9 cell lines. The cells were treated with doxorubicin in combination with 3 μM defactinib (n = 3 per point). **(K-N)** The tumor images taken in the end of the studies with 2 ovarian cancer animal models TOV21G (CDX), LD1-0032-361282 (PDX), 1 head and neck cancer PDX model HN-13-0286, and 1 small cell lung cancer PDX model LU5164. Data represent mean ± SEM.


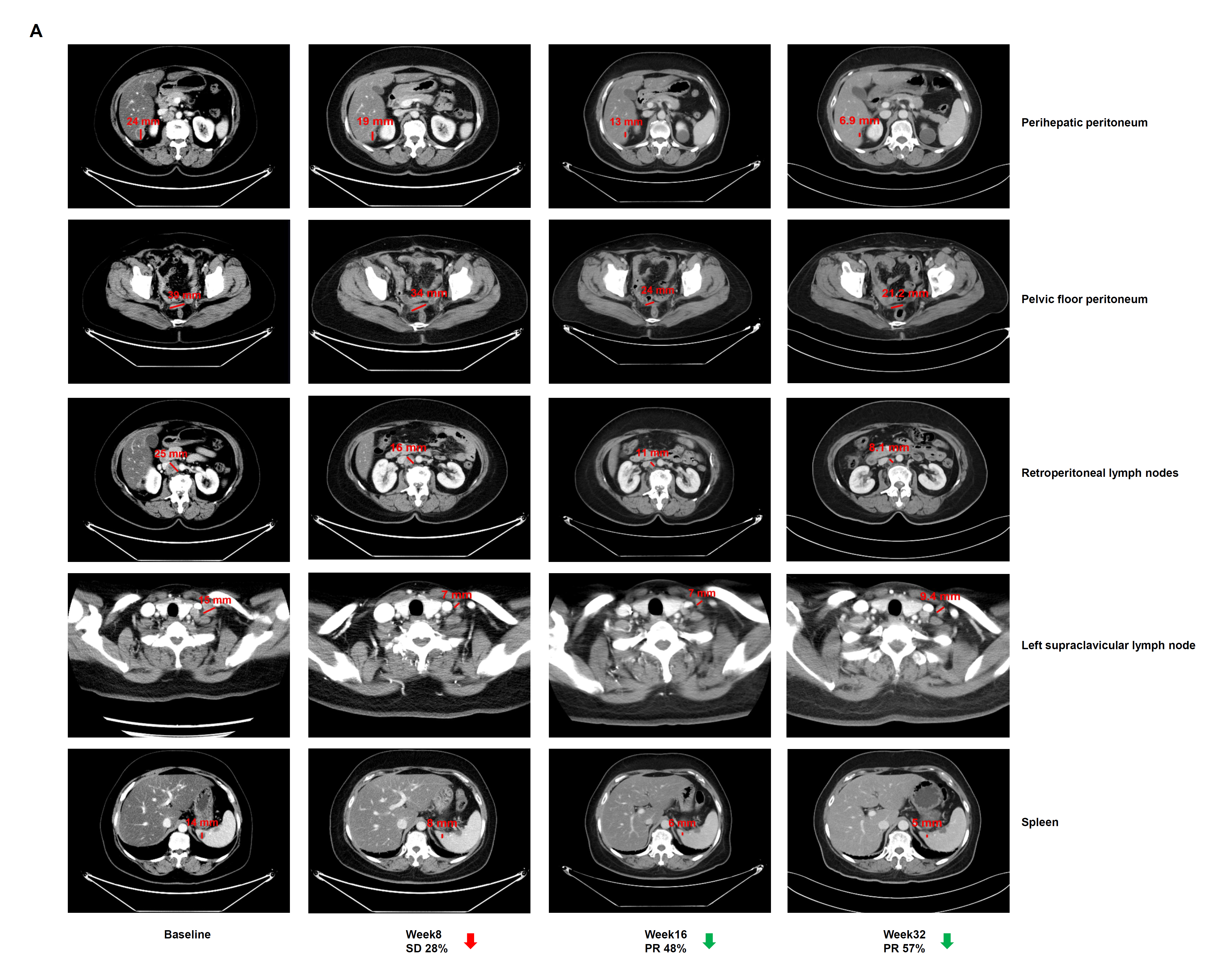


**Supplementary Figure 4:** The tumor images from No.4 patient.

**(A)** The tumor images of different lesions from No.4 patients. The imaging evaluation was performed on indicated timepoints. The patient had been evaluated with five different focuses of disease on perihepatic peritoneum, pelvic floor peritoneum, retroperitoneal lymph nodes, left supraclavicular lymph nodes, and spleen, respectively.


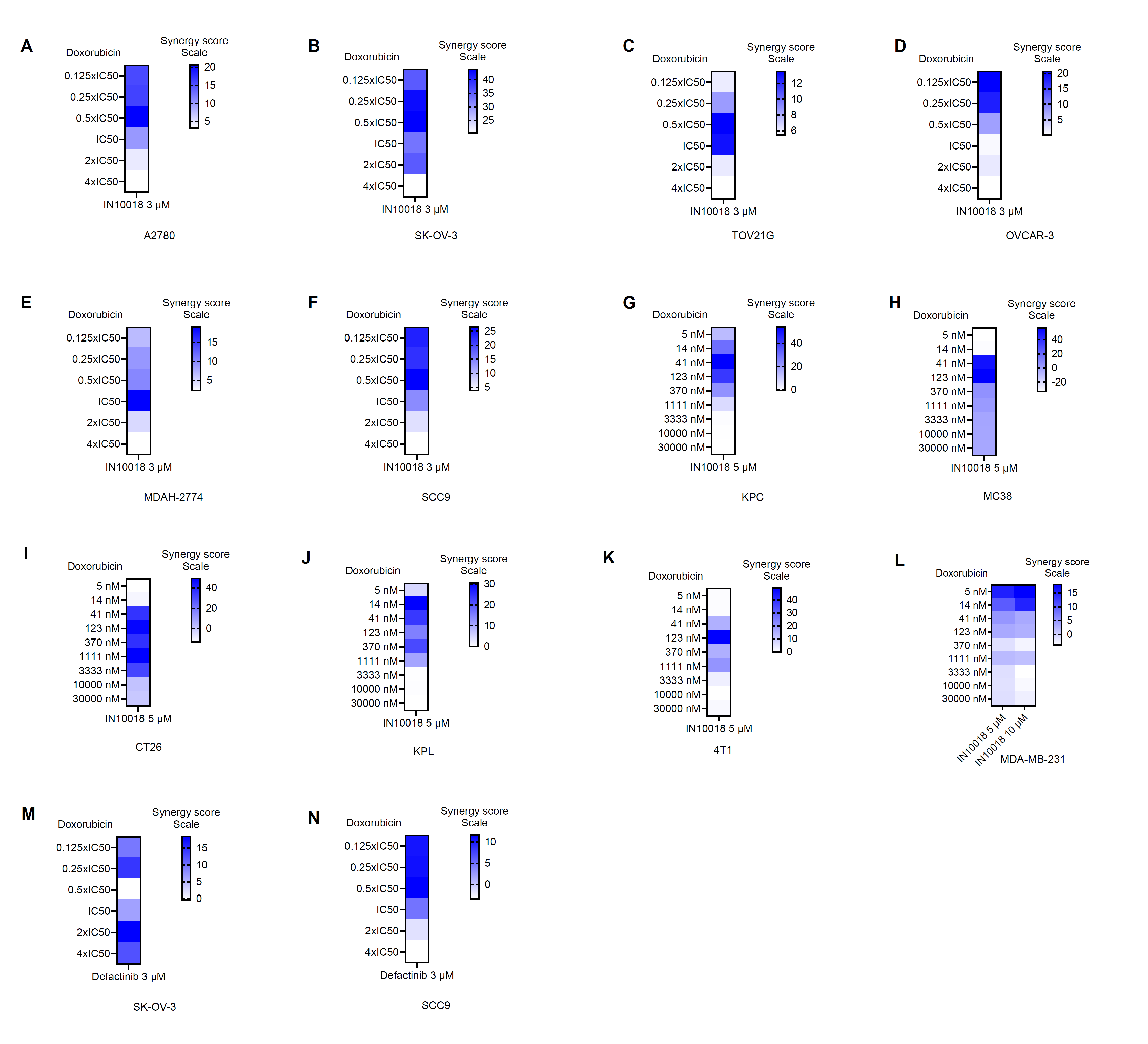


**Supplementary Figure 5:** The synergistic analysis for the combination treatment with doxorubicin and FAK inhibitors *in vitro*.

**(A-E)** The synergistic anticancer effects between doxorubicin and IN10018 in the treatment of ovarian cancer cell lines. The synergy score analysis was based on the data from Figure 2, B-F. **(F-L)** The synergistic effect analysis for the combination of doxorubicin and IN10018 in the treatment of other cancer types including head and neck cancer, NSCLC, pancreatic cancer, colorectal cancer, and breast cancer. The analysis was for the data from Figure S2, B-H. **(M-N)** The synergistic effect analysis for the combination of doxorubicin and defactinib for ovarian cancer cell line SK-OV-3 and head and neck cancer cell line SCC-9. All the synergy scores above were analyzed by synergy finder 2.0. The Synergy score > 10 represents synergistic antitumor effects between the 2 tested drugs.


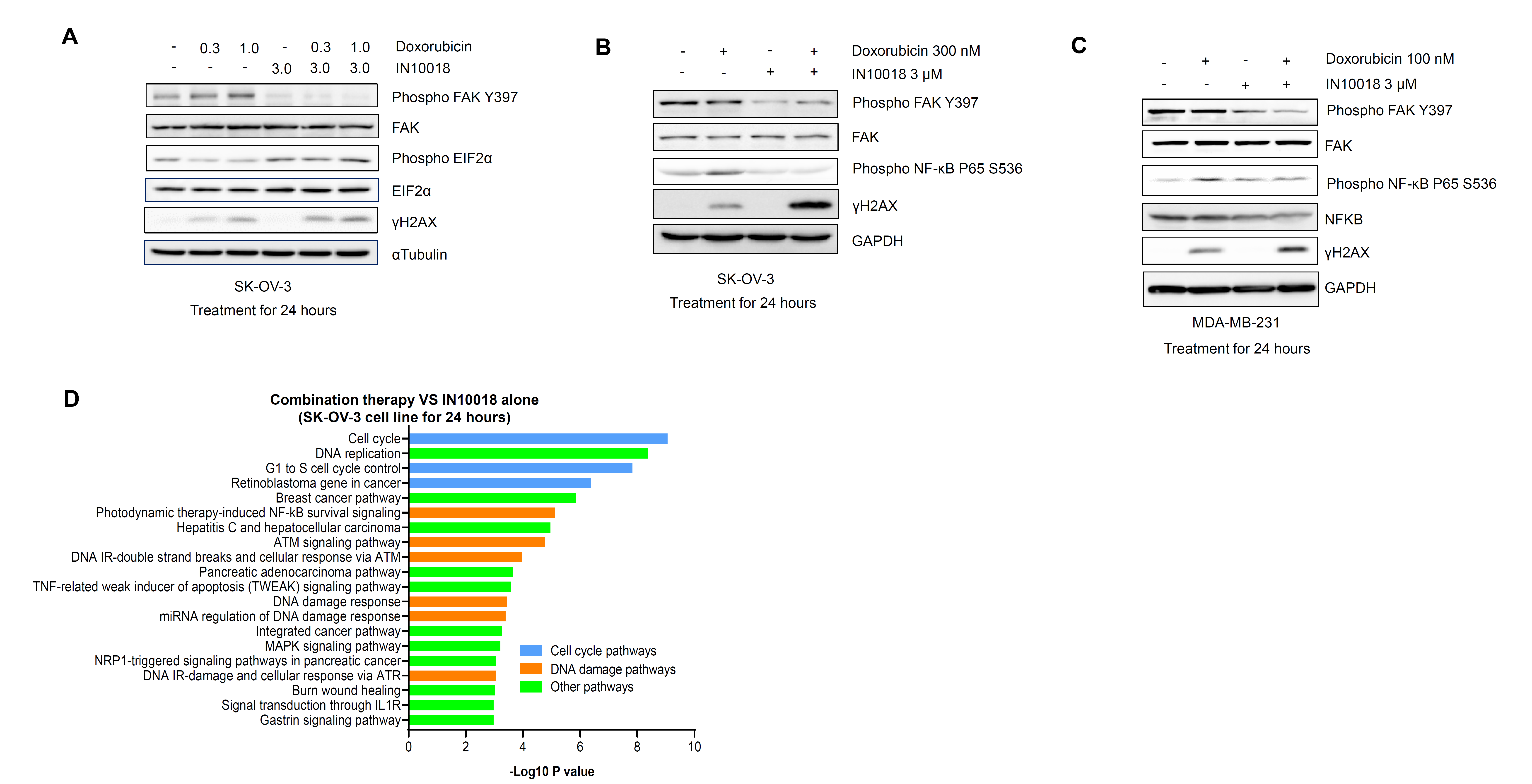


**Supplementary Figure 6:** The regulation of NF-κB and DNA damage signaling in part serves as the mechanism behind the drug combination effects of IN10018 and doxorubicin.

**(A-B)** IN10018 can decrease the enhancement of NF-κB signaling induced by doxorubicin and further strengthen the DNA damage exerted by doxorubicin on SK-OV-3 cells. **(C)** IN10018 can decrease the induced NF-κB signaling by doxorubicin and enhance DNA damage in combination with doxorubicin on the MDA-MB-231 cell line. **(D)** The most significantly altered signaling pathways upon combination treatment with IN10018 and doxorubicin through wiki pathway analysis of RNA sequencing. The items marked in blue indicate cell cycle-related signaling and those marked in red indicate DNA damage pathways.


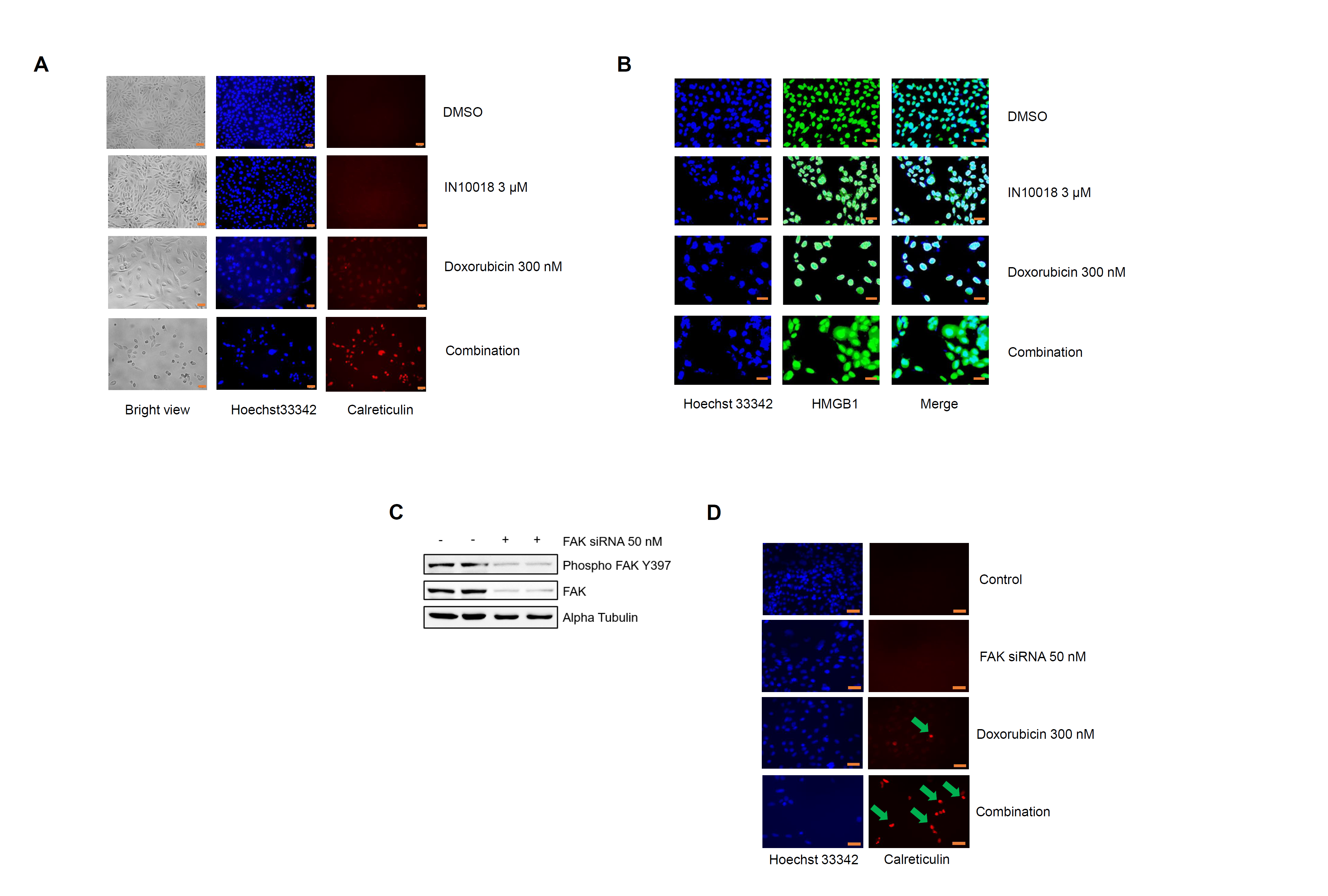


**Supplementary Figure 7:** The representative images for the immunofluorescence staining of cancer cells treated with the combination of FAK inhibition/knockdown and doxorubicin.

**(A-B)** Calreticulin, HMGB1, and Hoechst33342 staining for the SK-OV-3 cells treated with DMSO, 3 μM IN10018, 300 nM doxorubicin, and the drug combination. The cells were treated with the drugs for 48 hours. Then, they were fixed with 4% paraformaldehyde and stained for imaging analysis (Scale bar = 50 μm). **(C-D)** Knockdown of FAK protein level can enhance calreticulin exposure in combination with doxorubicin. After transfection with FAK siRNA for 24 hours, the SK-OV-3 cells were treated in combination with 300 nM doxorubicin for another 48 hours. Finally, the cells were collected for westernblot and calreticulin staining (Scale bar = 50 μm). Arrows indicate the positive staining cells.


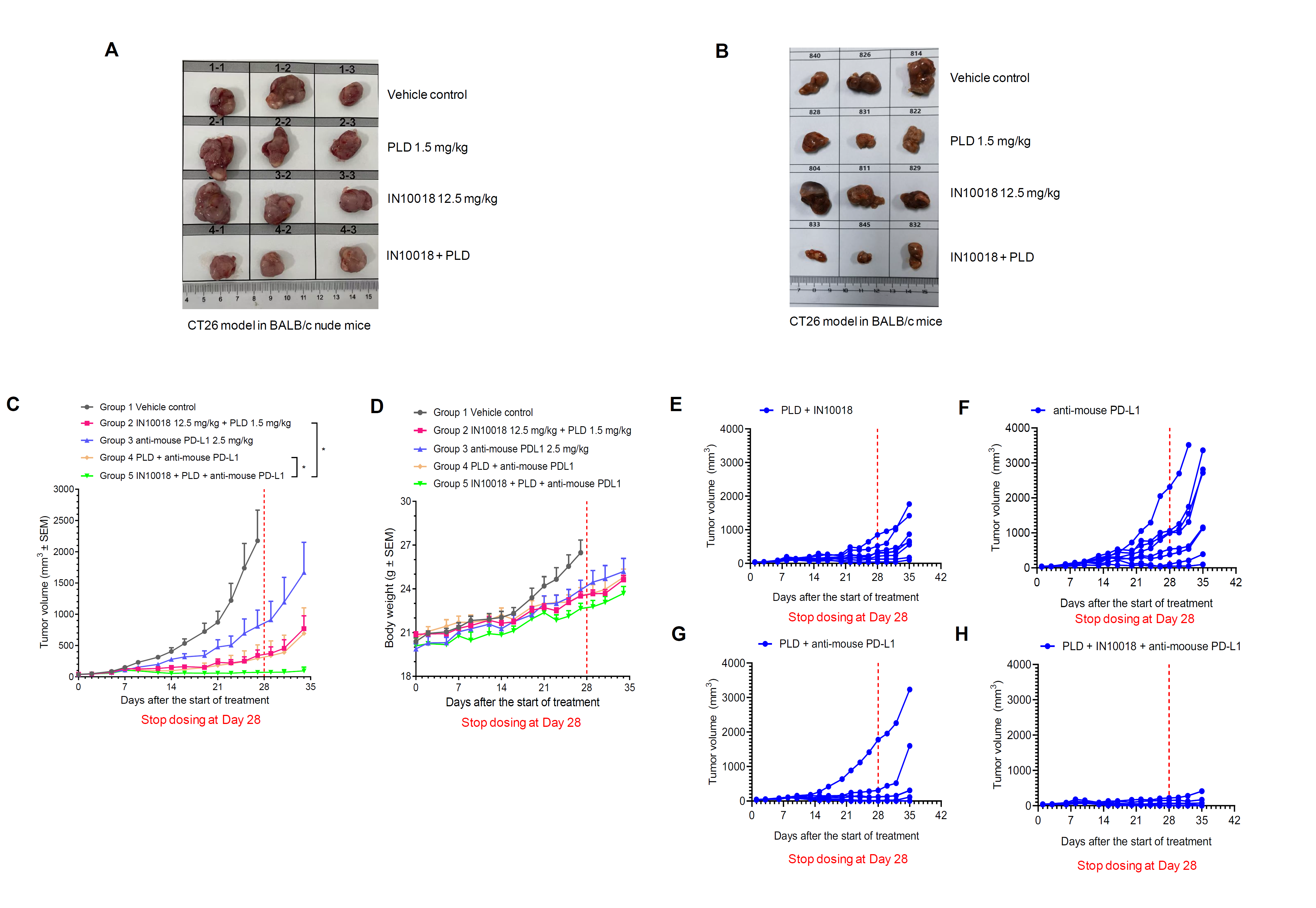


Supplementary Figure 8: The tumor images from the animal studies of CT26 model and the tumor growth inhibition for triple combination of PLD, IN10018, and PD-L1 blockade.

**(A-B)** The tumor images from the animal studies of CT26 model on BALB/c mice and BALB/c nude mice. **(C-D)** The triple combination testing including IN10018, PLD, and anti-mouse PD-L1 in the treatment of CT26 model generated on BALB/c mice. During the experiment, 12.5 mg/kg IN10018 was dosed orally once daily. 1.5 mg/kg PLD was dosed through tail vein injection once weekly. 2.5 mg/kg anti-mouse PD-L1 was intraperitoneally dosed twice a week. The treatment was stopped at day 28 (n = 8 per group). **(E-H)** The individual tumor growth curves from the triple combination study with IN10018, PLD, and anti-mouse PD-L1.


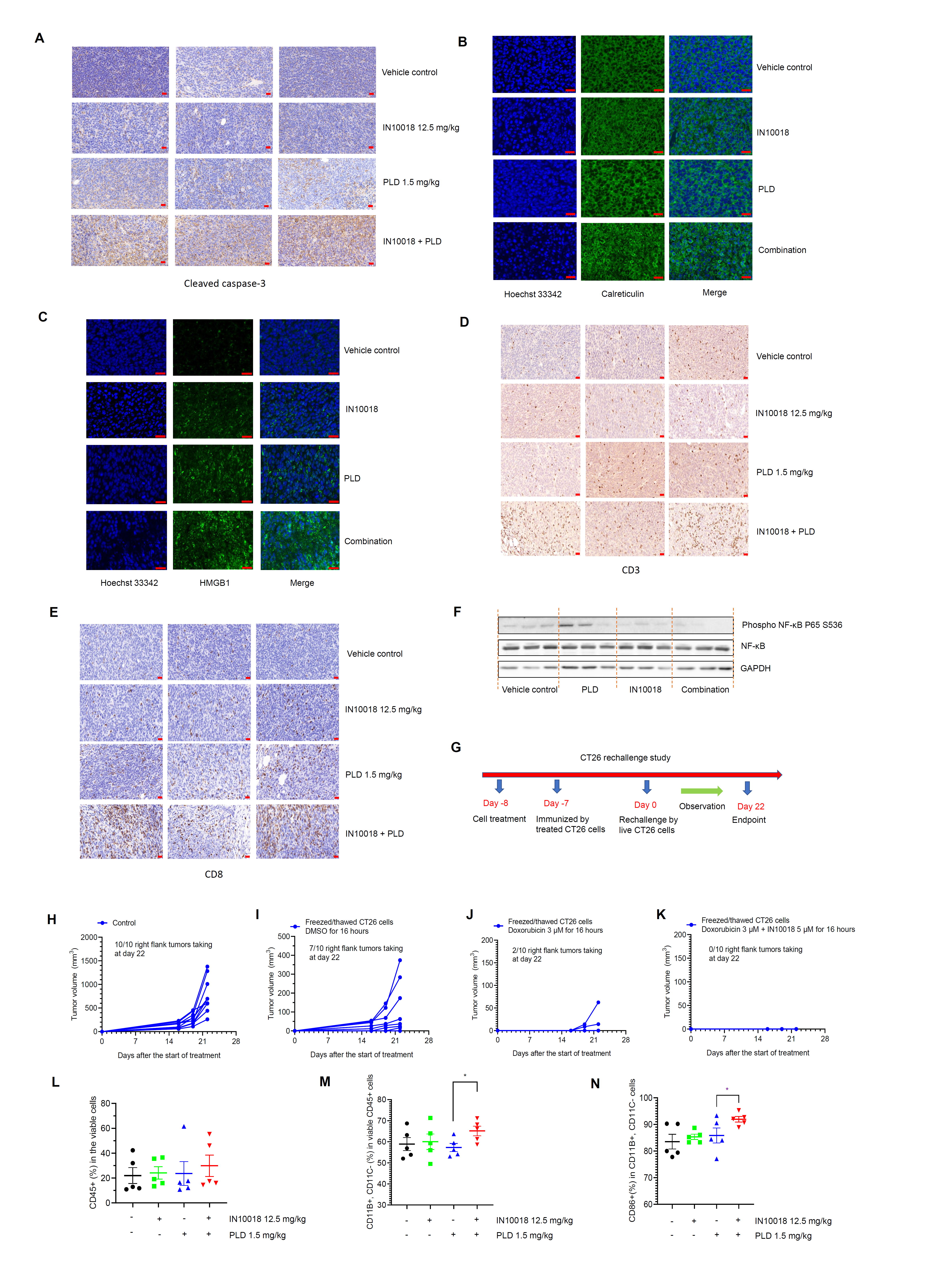


**Supplementary Figure 9:** The IHC, IF staining, and westernblot for the tumors from the combination test with IN10018 and PLD on CT26 syngeneic model and the CT26 rechallenging tests.

(A) Cleaved Caspase-3 IHC staining for the tumors from the efficacy study using the CT26 syngeneic model (Scale bar = 50 μm). **(B)** Calreticulin IF staining for the tumors from the efficacy study with CT26 syngeneic model (Scale bar = 20 μm). **(C)** HMGB1 IF staining for the tumors from the efficacy study with CT26 syngeneic model (Scale bar = 20 μm). **(D)** Westernblot for the tumors from the CT26 study. **(E)** IHC staining of CD3 for the tumors from CT26 efficacy study (Scale bar = 20 μm). **(F)** IHC staining of CD8 for the tumors from CT26 efficacy study (Scale bar = 20 μm). **(G-K)** CT26 tumor rechallenging study. The CT26 cancer cells were treated with DMSO, 3 μM Doxorubicin, and the combination of 3 μM Doxorubicin and 5 μM IN10018 for 16 hours, then the cells were conducted by 3 cycles of freezing/thawing and were injected to the left flank of the mice for immunization procedure. 7 days later, the mice were rechallenged by live CT26 cells. Tumor growth data were recorded for the evaluation of ICD potency *in vivo*. **(L-N)** FACs analysis for the tumors from the CT26 syngeneic model treated as the description above. The percentage of CD45+ cells were analyzed in all the viable tested cells from the dissociated tumor samples (J). The macrophages (K) and M1 macrophages (L) were represented by CD11B+, CD11C- in viable CD45+ cells and CD86+ in CD11B+, CD11C- cells, respectively (n = 5 per group). Data represent mean ± SEM. Statistical analysis was done using the unpaired student's T-test. *P < 0.05.

**Supplementary Figure 10:** The animal study data for the combination treatment of FAK inhibition and PLD in the treatment of 4T1 model.

**(A-B)** The animal study for the drug combination of IN10018 and PLD using 4T1 syngeneic model (n = 5 per group). **(C-I)** FACs analysis for the 4T1 tumors from (A-B). The samples were harvested on Day 13 post the start of treatment. DCs (CD11C+ percentages in viable CD45+ cells) (C), DCs maturation (CD80+ percentages in CD11C+ cells) (D), CD3+ T cells (CD3+ percentages in viable CD45+ cells) (E), CD4+ T cells (CD4+ percentages in CD3+ T cells) (F), CD8+ T cells (CD8+ percentages in CD3+ T cells) (G), and Treg cells (CD25+ and FOXP3+ percentages in CD4+ cells) (H) were filtered and analyzed using FACs. CD8/Treg ratio was calculated based on the FACs data (I) (n = 4 per group). Data represent mean ± SEM. Statistics analysis was done using the unpaired student's T-test. *P < 0.05, and **P < 0.01.


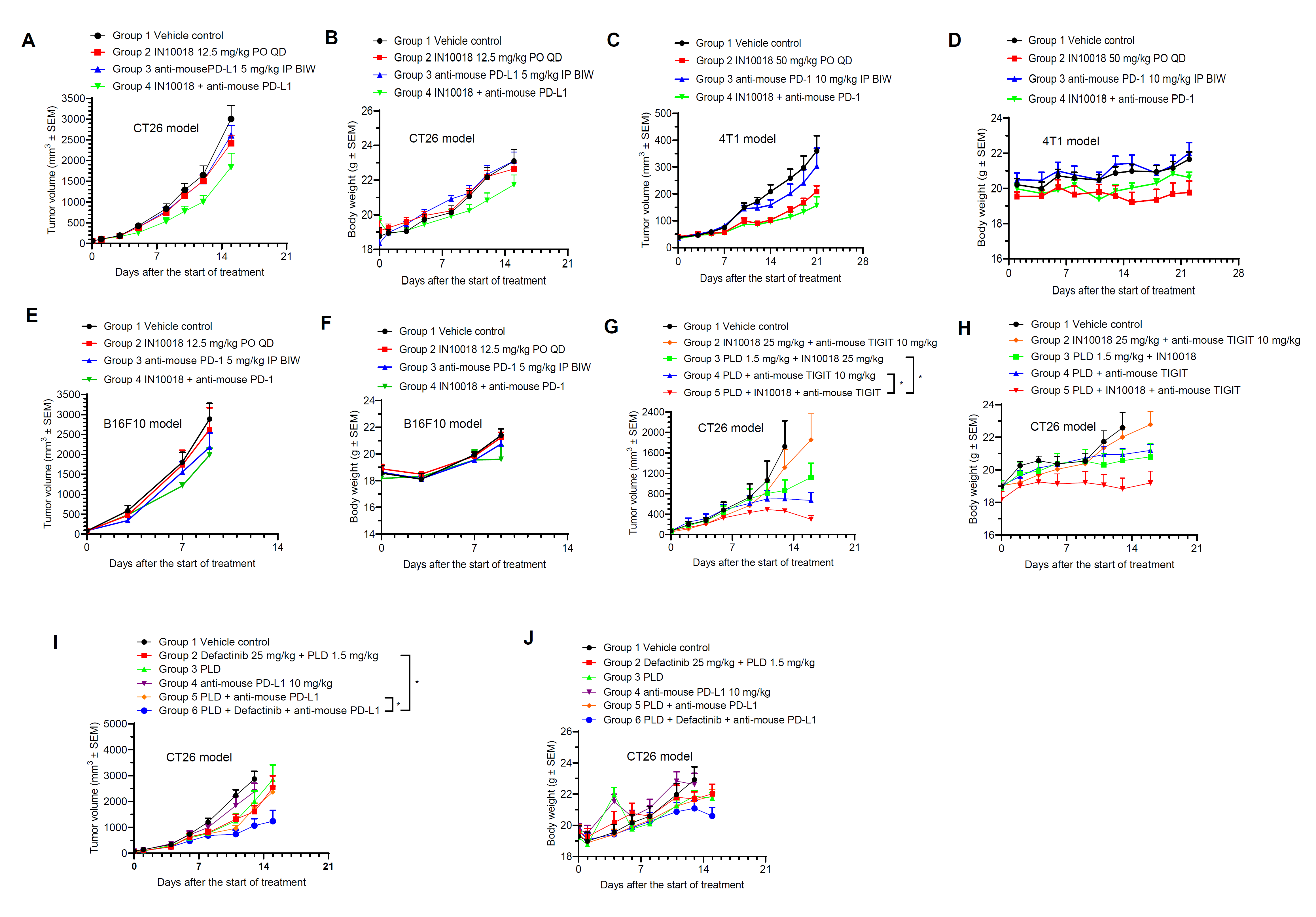


**Supplementary Figure 11:** The animal study data for the combination treatment of FAK inhibition, PLD, and immune checkpoint inhibition.

**(A-B)** The comparison of anti-tumor effect between anti-mouse PD-L1 and the combination of IN10018 and anti-mouse PD-L1 on CT26 syngeneic model. After grouping, anti-mouse PD-L1 was dosed by peritoneal injection twice a week. IN10018 was dosed by oral gavage once daily (n = 6 per group). **(C-D)** The doublet therapy of IN10018 and anti-mouse PD-1 on 4T1 syngeneic model. (n = 10 per group). **(E-F)** The combo-therapy of IN10018 and anti-mouse PD-1 on B16-F10 syngeneic model (n = 10 per group). **(G-H)** The triple combination test of IN10018, PLD and anti-mouse TIGIT on the CT26 syngeneic model. (n = 5 per group). **(I-J)** The triple combination test of defactinib, PLD, and anti-mouse PD-L1 in CT26 syngeneic model (n = 6 per group). In the above animal studies, IN10018 or defactinib was dosed via oral gavage once daily, PLD was dosed via tail vein injection once per week and anti-mouse PD-L1 or anti-mouse TIGIT was intraperitoneally dosed twice a week. Data represent mean ± SEM. Statistics analysis was done using one-way ANOVA. **P* < 0.05.

| **Patient ID** | **BOR** | **Tumor Shrinkage** | **Tumor Type** | **Histological Subtype** | **Prior Bevacizumab Use** | **Prior PARPi Use** | **Prior Treatment lines** | **PFI** | **ECOG** | **Gender** | **Race** | **Age** | **FIGO Stage** | **Baseline CA125** | **Target Lesion Size at baseline** |
| --- | --- | --- | --- | --- | --- | --- | --- | --- | --- | --- | --- | --- | --- | --- | --- |
| 1 | PD | -34% | Ovary Cancer | High-grade Serous | No | Yes | 2 | 44 | 0 | Female | Asian | 54 | Stage IVB | 227 | 44 |
| 2 | PD | -27% | Ovary Cancer | High-grade Serous | Yes | No | 1 | 114 | 0 | Female | Asian | 57 | Stage IVB | 3414 | 33 |
| 3 | PD | -23% | Ovary Cancer | High-grade Serous | Yes | No | 1 | 180 | 0 | Female | Asian | 55 | Stage III | 172 | 13 |
| 4 | SD | -1% | Ovary Cancer | High-grade Serous | No | Yes | 2 | 171 | 1 | Female | Asian | 64 | Stage IIIC | 243 | 34 |
| 5 | SD | 0% | Ovary Cancer | High-grade Serous | No | No | 1 | 78 | 0 | Female | Asian | 66 | Stage IV | 96 | 50 |
| 6 | SD | 3% | Ovary Cancer | High-grade Serous | No | No | 1 | 36 | 0 | Female | Asian | 49 | Stage IIIC | 506 | 30 |
| 7 | SD | 5% | Ovary Cancer | High-grade Serous | Yes | No | 1 | 86 | 0 | Female | Asian | 48 | Stage IVB | 505 | 85 |
| 8 | SD | 5% | Ovary Cancer | High-grade Serous | No | No | 1 | 92 | 0 | Female | Asian | 55 | Stage IIIC | 53 | 41 |
| 9 | SD | 6% | Ovary Cancer | High-grade Serous | No | No | 3 | 55 | 0 | Female | Asian | 50 | Stage IIIC | 61 | 49 |
| 10 | SD | 8% | Ovary Cancer | High-grade Serous | No | No | 4 | 157 | 0 | Female | Asian | 49 | Stage IIIB | 90 | 80 |
| 11 | SD | 9% | Ovary Cancer | High-grade Serous | No | No | 2 | 111 | 1 | Female | Asian | 56 | Unknown | 211 | 43 |
| 12 | SD | 11% | Ovary Cancer | High-grade Serous | No | No | 1 | 134 | 0 | Female | Asian | 50 | Stage IV | 32 | 63 |
| 13 | SD | 13% | Ovary Cancer | High-grade Serous | Yes | Yes | 1 | 177 | 1 | Female | Asian | 47 | Stage IIIC | 747 | 15 |
| 14 | SD | 20% | Ovary Cancer | High-grade Serous | No | No | 1 | 161 | 0 | Female | Asian | 58 | Stage IIIB | 776 | 36 |
| 15 | SD | 20% | Fallopian Tube Cancer | High-grade Serous | No | Yes | 4 | 112 | 1 | Female | Asian | 53 | Unknown | 110 | 99 |
| 16 | SD | 21% | Ovary Cancer | High-grade Serous | No | No | 4 | 143 | 0 | Female | Asian | 50 | Stage IV | 469 | 58 |
| 17 | PD | 24% | Ovary Cancer | High-grade Serous | No | No | 1 | 145 | 0 | Female | Asian | 54 | Stage IIIC | 2232 | 17 |
| 18 | SD | 28% | Ovary Cancer | High-grade Serous | No | No | 1 | 161 | 1 | Female | Asian | 52 | Stage IV | 18 | 36 |
| 19 | PR | 35% | Ovary Cancer | High-grade Serous | No | No | 1 | 166 | 0 | Female | Asian | 51 | Stage IIIC | 96 | 15 |
| 20 | PR | 39% | Ovary Cancer | High-grade Serous | No | No | 2 | 144 | 1 | Female | Asian | 66 | Stage IIIC | 78 | 79 |
| 21 | PR | 41% | Ovary Cancer | High-grade Serous | No | No | 1 | 71 | 1 | Female | Asian | 73 | Stage IVB | 15 | 112 |
| 22 | PR | 44% | Ovary Cancer | High-grade Serous | No | No | 2 | 133 | 0 | Female | Asian | 62 | Stage IIIC | 352 | 39 |
| 23 | PR | 46% | Ovary Cancer | High-grade Serous | No | No | 1 | 84 | 0 | Female | Asian | 44 | Stage IV | 51 | 36 |
| 24 | PR | 46% | Ovary Cancer | High-grade Serous | No | No | 1 | 137 | 0 | Female | Asian | 41 | Stage IIIC | 330 | 52 |
| 25 | PR | 47% | Ovary Cancer | High-grade Serous | No | No | 1 | 120 | 0 | Female | Asian | 57 | Stage IVA | 20 | 15 |
| 26 | PR | 48% | Ovary Cancer | High-grade Serous | No | No | 1 | 126 | 0 | Female | Asian | 65 | Stage IIIC | 265 | 42 |
| 27 | PR | 50% | Ovary Cancer | High-grade Serous | No | Yes | 1 | 135 | 1 | Female | Asian | 48 | Stage IV | 54 | 16 |
| 28 | PR | 50% | Ovary Cancer | High-grade Serous | No | No | 2 | 170 | 0 | Female | Asian | 65 | Stage IV | 7 | 26 |
| 29 | PR | 51% | Ovary Cancer | High-grade Serous | No | No | 2 | 107 | 1 | Female | Asian | 55 | Stage IVB | 45 | 33 |
| 30 | PR | 51% | Ovary Cancer | High-grade Serous | Yes | Yes | 3 | 36 | 0 | Female | Asian | 58 | Stage IV | 298 | 117 |
| 31 | PR | 52% | Ovary Cancer | High-grade Serous | No | No | 1 | 135 | 1 | Female | Asian | 70 | Stage IIIC | 377 | 27 |
| 32 | PR | 52% | Ovary Cancer | High-grade Serous | Yes | No | 2 | 120 | 0 | Female | Asian | 50 | Stage IV | 1059 | 48 |
| 33 | PR | 53% | Ovary Cancer | High-grade Serous | No | No | 1 | 72 | 0 | Female | Asian | 57 | Stage IIIC | 133 | 68 |
| 34 | PD | 58% | Primary Peritoneum Cancer | High-grade Serous | No | Yes | 1 | 149 | 0 | Female | Asian | 67 | Stage IVB | 593 | 33 |
| 35 | PR | 63% | Ovary Cancer | High-grade Serous | Yes | Yes | 5 | 71 | 1 | Female | Asian | 65 | Stage IIIC | 387 | 46 |
| 36 | PR | 65% | Ovary Cancer | High-grade Serous | No | No | 1 | 140 | 0 | Female | Asian | 52 | Stage IVB | 942 | 81 |
| 37 | PR | 67% | Ovary Cancer | High-grade Serous | No | No | 1 | 146 | 0 | Female | Asian | 59 | Stage IIIC | 64 | 30 |
| 38 | PR | 72% | Ovary Cancer | High-grade Serous | No | No | 2 | 103 | 0 | Female | Asian | 57 | Stage IIIC | 316 | 36 |
| 39 | PR | 73% | Ovary Cancer | High-grade Serous | No | No | 1 | 125 | 0 | Female | Asian | 50 | Stage IIIC | 434 | 165 |
| 40 | PR | 77% | Ovary Cancer | High-grade Serous | No | No | 1 | 35 | 0 | Female | Asian | 46 | Stage IIIA1 | 13 | 47 |
| 41 | PR | 78% | Ovary Cancer | High-grade Serous | No | No | 1 | 30 | 1 | Female | Asian | 57 | Stage IV | 19 | 23 |
| 42 | PR | 83% | Ovary Cancer | High-grade Serous | No | No | 1 | 53 | 0 | Female | Asian | 60 | Stage IVB | 279 | 12 |

**Supplementary Table 1:** The characteristics, treatments, and responses of the efficacy-evaluable patients in the clinical trial with dual regimen of PLD and IN10018 in the treatment of PROC.

As of the cutoff date of 31 May 2022, A total of 50 PROC patients were enrolled for this single-arm clinical trial. Anti-cacner effects were evaluable in 42 patients. The abbreviations used in the table are as follows. ID: Identity; BOR: Best Overall Response; PD: Progressive Disease; SD: Stable Disease; PR: Partial Response; PARPi: PARP inhibitor; PFI: Progression Free Interval; ECOG: Eastern Cooperative Oncology Group; FIGO: The International Federation of Gynecology and Obstetrics.

| **Subjects with AEs** | **IN10018 + PLD** |
| --- | --- |
|  | **N (%)** |
| Number of Subjects who took at least one dose | 50 (100.0) |
| Subjects with any AE | 48 (96.0) |
| Subjects with IN10018-related AEs | 47 (94.0) |
| Subjects with SAEs | 11 (22.0) |
| Subjects with IN10018-related SAEs | 5 (10.0) |
| Subjects with CTCAE Grade ≥3 AEs | 25 (50.0) |
| Subjects with IN10018 related Grade ≥3 AEs (Judged by Investigators) | 9 (18.0) |
| Subjects with AEs leading to death | 2 (4.0) |
| Subjects with IN10018-related AEs leading to death | 0 |
| Subjects with AESI | 5 (10.0) |
| Subjects with AEs leading to IN10018 dose reduction | 3 (6.0) |
| Subjects with AEs leading to IN10018 dose discontinuation | 2 (4.0) |

**Supplementary Table 2:** The AEs summary for the clinical trial.

As of the cutoff date of the dual regimen trial, A total of 50 PROC patients were enrolled and have records of AEs. For the triplet regimen trial, a total of 23 PROC patients were enrolled and have assessment of AEs. The abbreviations used in the table were as follows. AEs: Adverse Events; SAE: Serious Adverse Events; CTCAE: Common Terminology Criteria for Adverse Events; AESI: Adverse Event of Special Interest.

| **Items** | **Sequences** |
| --- | --- |
| FAK siRNA | GGUCAGAGAUACUUCUUAATTUUAAGAAGUAUCUCUGACCTT |
| Anti-mouse TIGIT | >10A7 VK  DIVMTQSPSSLAVSPGEKVTMTCKSSQSLYYSGVKENLLAWYQQKPGQSPKLLIYYASIRFTGVPDRFTGSGSGTDYTLTITSVQAEDMGQYFCQQGINNPLTFGDGTKLEIK  >10A7 VH  EVQLVESGGGLTQPGKSLKLSCEASGFTFSSFTMHWVRQSPGKGLEWVAFIRSGSGIVFYADAVRGRFTISRDNAKNLLFLQMNDLKSEDTAMYYCARRPLGHNTFDSWGQGTLVTVSS |

**Supplementary Table 3:** The sequences of FAK siRNA and anti-mouse TIGIT used in the study.

| **Antibody** | **Vendor** | **Host species** | **Dilution factor** | **Catalog No.** |
| --- | --- | --- | --- | --- |
| FITC anti-mouse CD11C | Biolegend | Armenian Hamster | 1:400 | 117306 |
| PE anti-mouse/human CD11B | Biolegend | Armenian Hamster | 1:400 | 101208 |
| CD3 | Abcam | Rabbit | 1:400 | ab16669 |
| Brilliant Violet 421™ anti-mouse CD45 | Biolegend | Rat | 1:400 | 103134 |
| EIF2α | CST | Rabbit | 1:1000 | 5324S |
| CD8α | CST | Rabbit | 1:400 | 98941S |
| PE anti-mouse CD80 | Biolegend | Armenian Hamster | 1:400 | 104708 |
| APC anti-mouse CD86 | Biolegend | Rat | 1:400 | 105114 |
| APC anti-mouse CD40 | Biolegend | Rat | 1:400 | 124612 |
| Brilliant Violet 421™ anti-mouse I-A/I-E (MHCII) | Biolegend | Rat | 1:400 | 107632 |
| Brilliant Violet 605™ anti-mouse CD3 | Biolegend | Rat | 1:400 | 100237 |
| Percp-cy5.5 anti-mouse CD8α | Biolegend | Rat | 1:400 | 100734 |
| Brilliant Violet 510™ anti-mouse CD4 | Biolegend | Rat | 1:400 | 100449 |
| Brilliant Violet 421™ anti-mouse CD25 | Biolegend | Rat | 1:400 | 101923 |
| AF647 anti-mouse FOXP3 | Biolegend | Rat | 1:400 | 126408 |
| Phospho-EIF2α S51 | CST | Rabbit | 1:1000 | 3398S |
| Calreticulin | CST | Rabbit | 1:1000 | 4691T |
| HRP-conjugated alpha Tubulin | Proteintech | Mouse | 1:5000 | HRP-66031 |
| HRP-conjugated GAPDH | Proteintech | Mouse | 1:5000 | HRP-60004 |
| EIF2α | CST | Rabbit | 1:2000 | 5324S |
| HMGB1 | CST | Rabbit | 1:2000 | 4695S |
| GRP94 | HUABIO | Rabbit | 1:1000 | ER1511-5 |
| NF-κB | HUABIO | Rabbit | 1:1000 | ET1603-12 |
| Cleaved Caspase-3 | CST | Rabbit | 1:2000 | 9664S |
| γH2AX | CST | Rabbit | 1:1000 | 14074S |
| Phospho NF-κB P65 S536 | CST | Rabbit | 1:1000 | 3033S |
| Phospho EIF2 alpha S51 | CST | Rabbit | 1:2000 | 3597S |
| Phospho FAK Y397 | Thermo fisher scientific | Rabbit | 1:1000 | 44-624G |
| Phospho-Histone H2A.X S139 (γH2AX) | CST | Rabbit | 1:2000 | 9718S |
| Anti-rabbit IgG (H+L), F(ab')2 Fragment (Alexa Fluor® 647 Conjugate) | CST | Goat | 1:1000 | 4414S |
| Anti-mouse IgG (H+L), F(ab')2 Fragment (Alexa Fluor® 488 Conjugate) | CST | Goat | 1:1000 | 4408S |
| Goat anti-rabbit IgG (H+L) secondary antibody HRP | Invitrogen | Goat | 1:5000 | 31460 |
| Goat anti-mouse IgG (H+L) secondary antibody HRP | Invitrogen | Goat | 1:5000 | 31430 |
| *Invivo*MAb anti-mouse PD-1 (CD279) | BioXcell | Rat | - | BE0146 |
| *Invivo*MAb anti-mouse PD-L1 (B7-H1) | BioXcell | Rat | - | BE0101 |
| Anti-mouse TIGIT | In house | Mouse | - | - |

**Supplementary Table 4:** The antibodies used in the study.
